# Rapid Identification of Coumarins from *Micromelum falcatum* by UPLC-HRMS/MS and Targeted Isolation of Three New Derivatives

**DOI:** 10.3390/molecules190915042

**Published:** 2014-09-19

**Authors:** Eirini Kouloura, Eirini Danika, Sothea Kim, Mélanie Hoerlé, Muriel Cuendet, Maria Halabalaki, Leandros A. Skaltsounis

**Affiliations:** 1 Laboratory of Pharmacognosy & Natural Products Chemistry, Faculty of Pharmacy, University of Athens, Panepistimioupoli, Zografou, 15771 Athens, Greece; E-Mails: eikouloura@pharm.uoa.gr (E.K.); irinidan@pharm.uoa.gr (E.D.); skaltsounis@pharm.uoa.gr (L.A.S.); 2 Joint Laboratory of Phytochemistry, University of Health Sciences, 73 Bd Monivong, Phnom Penh 12203, Cambodia; E-Mail: kimsothea@uhs.edu.kh; 3 School of Pharmaceutical Sciences, University of Geneva, University of Lausanne, Quai Ernest-Ansermet 30, CH-1211 Geneva 4, Switzerland; E-Mails: melanie.hoerle@gmail.com (M.H.); Muriel.Cuendet@unige.ch (M.C.)

**Keywords:** *Micromelum falcatum*, Rutaceae, 7-oxygenated coumarins, LC-MS, LTQ-Orbitrap, microfalcrin, microcoumaririn, micromelosidester, anti-inflammatory activity, NF-κB, NO production

## Abstract

*Micromelum falcatum*, a medicinal plant of the Rutaceae family, has been used in the Traditional Chinese Medicine (TCM) mainly against colds and rheumatoid arthritis. Despite its traditional use the association of its constituents with possible anti-inflammatory activity has not been explored. During this study, a rapid UPLC-ESI(+)-HRMS method was developed for the profiling of *M. falcatum* leave extracts and the targeted isolation of coumarin constituents. Based on chromatographic, spectroscopic and spectrometric features several 7-oxygenated coumarin derivatives were detected. After targeted isolation, eight coumarins, among them three new natural products, namely microfalcrin, microcoumaririn and micromelosidester, were purified using semi-preparative HPLC and unambiguously identified by 1 and 2D NMR. Furthermore, important spectrometric characteristics were revealed based on the HRMS and HRMS/MS spectra of the isolated 7-oxygenated coumarins facilitating their identification in complex mixtures. Finally, the anti-inflammatory properties of the extracts and representative compounds were evaluated by measuring the inhibition of the pro-inflammatory mediator NF-κB induction and nitric oxide (NO) production.

## 1. Introduction

*Micromelum falcatum* of the Rutaceae family is a shrub widely distributed in tropical and subtropical regions of the Indochina peninsula [1]. In Traditional Chinese Medicine (TCM) the leaves have been used to treat fevers, rhinorrhea and as an antidote for intoxications. Infusions of the leaves have also been used against headaches and menstrual disorders, while the bark of the plant applied externally has been used to heal wounds and infections. Both leaves and barks have been reported as remedies for rheumatism [2,3,4]. Previous studies on *M. falcatum* have revealed the presence of several groups of secondary metabolites such as derivatives of dihydrocinnamic acid [5] and most importantly, alkaloids and coumarins. Specifically, numerous alkaloids belonging to the quinolone, quinoldione [6], indole and carbazole [7] groups have been isolated from the bark of the plant. In addition, several 6- and 8-prenylated derivatives of 7-oxygenated and specifically 7-methoxylated coumarins have been reported from the bark and the leaves of *M. falcatum* [5,7,8,9].

Coumarins are considered important pharmacological agents abundantly present in the Rutaceae family [10]. The main pharmacological assays used to evaluate the biological properties of coumarins isolated from *Micromelum* species have been focused on their cytotoxicity and have demonstrated moderate activity against several cancerous cell lines [9,11]. However, the traditional use of *M. falcatum* implies a potential anti-inflammatory activity which has not been examined up to now, despite the fact that coumarins have been proven prominent compounds that attenuate excessive and prolonged nitric oxide (NO) generation at inflammatory sites [12], and regulate the NF-κB pathway [13].

As part of our investigations for new natural anti-inflammatory agents, a phytochemical study of *M. falcatum* leaves was performed. The HPLC-DAD and UPLC-ESI(+)-HRMS and HRMS/MS profiling of different extracts of *M. falcatum* revealed useful spectroscopic and spectrometric features which enabled the detection of numerous coumarin derivatives and led to a targeted isolation thereof. In total, eight 7-methoxylated coumarins, among them the three new natural products microfalcrin (**1**), microcoumaririn (**2**), micromelosidester (**3**) and the known ones micromeloside A (**4**) [8], microminutin (**5**) [11], micromelin (**6**) [14], micromarin B (**7**) [15] and micromarin A (**8**) [15] were isolated and structurally elucidated. In addition, the inhibition of the NO synthase and the pro-inflammatory agent NF-κB by representative extracts and compounds was explored.

## 2. Results and Discussion

### 2.1. HPLC-DAD and UPLC-ESI(+)-HRMS/MS Profiling of Micromelum falcatum Extracts

In order to selectively detect coumarin derivatives, the profiling of different *M. falcatum* extracts was performed using HPLC-DAD and UPLC-ESI(+)-HRMS/MS instruments. Two different extraction methods were followed and the obtained extracts were compared based on chromatographic (Rt), spectral (UV) and spectrometric (HR full scan and MS/MS) features. In particular, a part of the plant material was extracted using DCM, MeOH and H_2_O successively as extraction solvents. In parallel, since *Micromelum* is reported to contain alkaloids, another part of the plant material was extracted following a specific protocol for alkaloids. This protocol involves the extraction of the plant material with EtOAc and then alkalinization of the plant residue and successive extraction with EtOAc and MeOH. The derived extracts were partitioned using HCl solution and after a second alkalinization step organic (EtOAc_A, MeOH_A) and aqueous (EtOAc_B, MeOH_B) fractions were obtained, respectively (see Section 3.3. for further details). Since, this study was focused on the identification of coumarin constituents the alkaloid content was not further investigated.

All the extracts were analyzed by HPLC-DAD (Figure S1) and the comparison of their chromatograms revealed that the second extraction approach was qualitatively more efficient regarding the secondary metabolite content. As expected, the organic fractions (EtOAc_A, MeOH_A) were found more enriched in lipophilic compounds while the more polar constituents were concentrated in the aqueous fractions (EtOAc_B, MeOH_B) (Figure S2). In addition, concerning the classical extraction approach, the DCM extract was found more rich compared to the MeOH and H_2_O extracts which were found less abundant in secondary metabolites. It is worth noting that in all extracts a dominant metabolite was observed at 21 min (Figure S1) with characteristic UV absorption maxima at 206 and 321 nm indicative of 7-oxygenated coumarins. Based on UV spectra, all extracts were screened and mainly in DCM, EtOAc, EtOAc_A, EtOAc_B, MeOH_A and MeOH_B extracts peaks providing similar absorption maxima were found.

**Figure 1:**
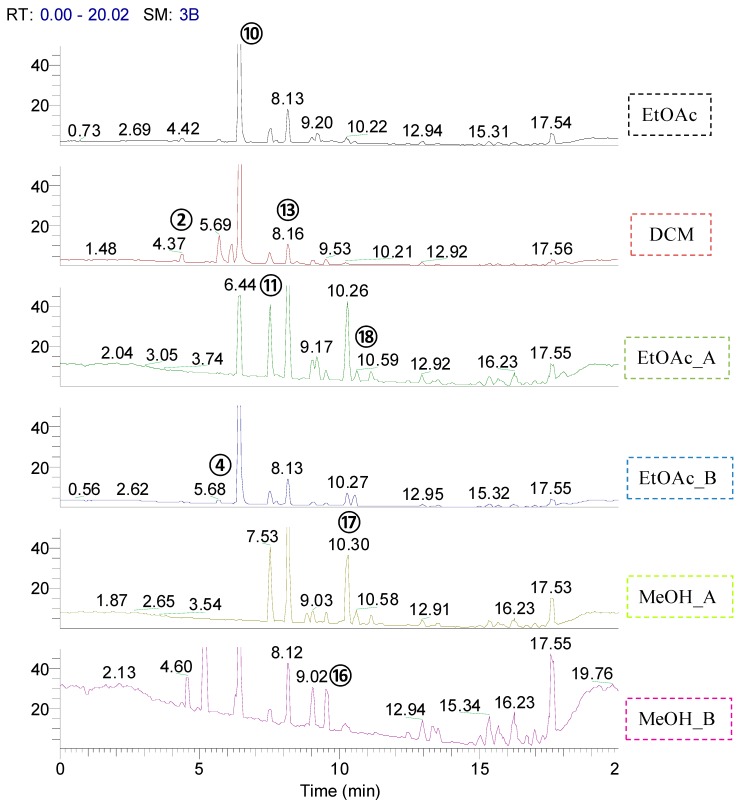
Base peak chromatograms of each extract obtained from *M. falcatum* in positive ionization mode.

Thereafter, the aforementioned extracts were analyzed by UPLC-ESI(+)-HRMS/MS in order to obtain characteristic spectrometric data for coumarin compounds and diagnostic fragment ions which could facilitate their detection in complex mixtures. To the best of our knowledge, no previous data have been reported concerning the analysis of 7-oxygenated coumarins by UPLC-ESI(+)-HRMS/MS. The LC-MS-based profiling of *M. falcatum* extracts revealed several peaks in the base peak chromatograms (Figure 1). In addition, the data dependent acquisition method using dynamic exclusion enabled the collection of numerous HRMS/MS spectra for each resolved peak. Taking advantage of the accurate mass measurements and the high resolution of the Orbitrap analyzer, a specific strategy was followed [16]. Initially, descriptors such as the suggested elemental composition (EC), the RDB eq. values and the Δm (ppm) between the theoretical and measured values were calculated for each detected peak. For the prioritization of the suggested ECs, analysis of the reported 7-oxygenated coumarins from *Micromelum* species led to the determination of certain thresholds. Assuming that 7-oxygenated coumarins present in the different extracts are substituted structures of the simple 7-oxygenated coumarin, specific limitations were set. In particular, a minimum number of 10 C atoms, seven H atoms and three O atoms were selected and a RDB eq. value greater than 7 was defined. The Δm was set ≤ 3 ppm since the analysis was performed using the Orbitrap analyzer routinely operating in 1–2 ppm accuracy window, at low masses [17].

Armed with the information from the UV spectra as a starting point, the most abundant peak at 6.42 min (21 min in the HPLC-DAD chromatogram) which presented characteristic absorption maxima of a 7-oxygenated coumarin, was analyzed. The main ion in the full scan spectrum revealed the presence of an adduct ion with sodium [M+Na]^+^ while the corresponding [M+H]^+^ wasn’t detected. Based on this observation and the abovementioned criteria, all the peaks satisfying both conditions were defined and listed. Scrutinizing the literature, in combination with UPLC-ESI(+)-HRMS/MS data, 10 known coumarins were detected and eight possibly new coumarins were proposed in all selected extracts (Table 1).

**Table 1:** 7-Oxygenated coumarin derivatives detected in *M. falcatum* extracts based on literature and UPLC-ESI(+)-HRMS/MS data.

No	Rt (min)	[M+Na]^+^	Molecular Formula	RDB	Delta (ppm)	MS/MS (% Intensity)	Suggested Compounds (Annotation)	Extract
1	4.10	315.0842	C_15_H_16_O_6_Na	7.5	1.018	297.0737 (24)	Unknown (Coumarin with aliphatic chain)	EtOAc, DCM, EtOAc_A
						285.0737 (5)		
						227.0317 (100)		
2	4.37	315.0842	C_15_H_16_O_6_Na	7.5	0.922	297.0737 (17)	Unknown (Microfalcrin, **1**)	EtOAc, DCM, EtOAc_A, EtOAc_B
						285.0737 (3)		
						227.0316 (100)		
3	5.20	357.0947	C_17_H_18_O_7_Na	8.5	0.633	297.0736 (100)	Micromeloside D	DCM, EtOAc_A
4	5.68	297.0737	C_15_H_14_O_5_Na	8.5	1.196		Micromeloside A/Hopeyhopin (Micromeloside A, **4**)	EtOAc, DCM, EtOAc_A, EtOAc_B
5	5.88	357.0948	C_17_H_18_O_7_Na	8.5	1.053	297.0737 (56)	Micromeloside D	DCM, EtOAc_A
						227.0317 (100)		
6	6.06	299.0893	C_15_H_16_O_5_Na	7.5	0.887	281.0788 (100)	Hydramicromelin A/Murrangatin	EtOAc
						269.0788 (81)		
7	6.12	311.0529	C_15_H_12_O_6_Na	9.5	1.095		Micromelin (Microcoumaririn, **2**)	DCM, EtOAc_A
8	6.29	301.1050	C_15_H_18_O_5_Na	7.5	1.312	283.0945 (100)	Unknown	DCM, EtOAc_A
9	6.30	315.0843	C_15_H_16_O_6_Na	7.5	1.113	297.0852 (100)	Unknown	EtOAc, EtOAc_B
10	6.42	295.0579	C_15_H_12_O_5_Na	9.5	0.662		Microminutin(Microminutin, **5**)	EtOAc, DCM, EtOAc_A, EtOAc_B, MeOH_A, MeOH_B
11	6.55	311.0527	C_15_H_12_O_6_Na	9.5	1.053		Micromelin (Micromelin, **6**)	EtOAc, DCM, EtOAc_B
12	7.54	399.1416	C_20_H_24_O_7_Na	8.5	0.365	297.0736 (100)	Micromarin B/C	EtOAc, DCM, EtOAc_A, EtOAc_B, MeOH_A, MeOH_B
13	7.73	399.1416	C_20_H_24_O_7_Na	8.5	0.516	297.0736 (100)	Micromarin B/C	EtOAc, EtOAc_B
14	8.17	399.1416	C_20_H_24_O_7_Na	8.5	0.516	381.1312 (5)	Micromarin B/C (Micromarin B, **7**)	EtOAc, DCM, EtOAc_A, EtOAc_B, MeOH_A, MeOH_B
						297.0737 (75)		
						227.0317 (100)		
15	8.44	343.1156	C_17_H_20_O_6_Na	7.5	1.138	315.0843 (100)	Micromeloside B	DCM, EtOAc_A, EtOAc_B
						297.0737 (60)		
16	8.81	413.1573	C_21_H_26_O_7_Na	8.5	0.522	311.0894 (100)	Micromeloside C	EtOAc_A, MeOH_A
17	9.52	409.1624	C_22_H_26_O_6_Na	9.5	0.465	289.1050 (100)	Unknown (Coumarin with isovaleryl chain)	EtOAc, DCM, EtOAc_A, EtOAc_B, MeOH_A, MeOH_B
18	10.27	381.1311	C_20_H_22_O_6_Na	9.5	0.710	279.0630 (100)	Micromarin A (Micromelosidester, **3**)	EtOAc, DCM, EtOAc_B, MeOH_A
19	10.55	381.1312	C_20_H_22_O_6_Na	9.5	0.893	353.1363 (28)	Micromarin A (Micromarin A, **8**)	EtOAc, EtOAc_B, MeOH_A
						279.0630 (100)		
						257.0812 (41)		

Based on these data targeted isolation of representative known and unknown coumarins were followed. Furthermore, the purified coumarins were used as reference compounds using the direct infusion method in order to investigate their spectrometric behavior and characteristics.

### 2.2. Isolation and Structure Elucidation of 7-Oxygenated Coumarin Derivatives

**Figure 2:**
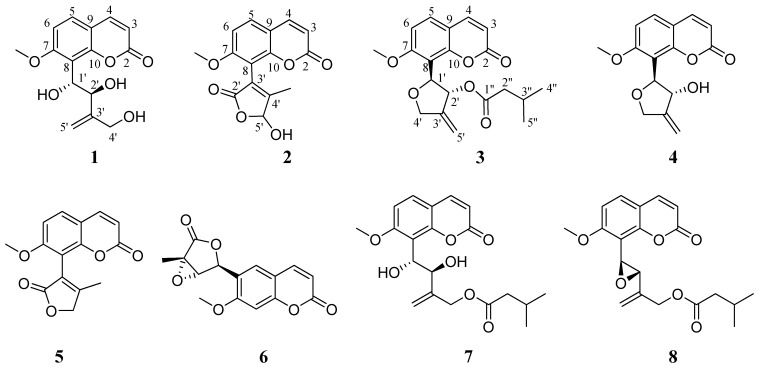
Structures of compounds **1**–**8**.

Based on the profiling of *M. falcatum* extracts, specific peaks were selected for targeted isolation of representative coumarins. The most promising extracts (DCM/EtOAc/EtOAc_A) were subjected to HPLC-DAD semi-preparative isolation. Specifically, eight peaks were targeted and finally five known coumarins of the genus together with three new coumains were purified (Figure 2). For structure determination and unambiguous identification 1 and 2 D NMR spectra were acquired verifying in all cases the initial predictions.

Compound **1** (peak 2, Rt = 4.37 min) was isolated as a white amorphous powder. The UV spectrum in methanol revealed the characteristic absorption maxima for 7-oxygenated coumarins at 206 and 319 nm [5]. Its molecular formula was deduced as C_15_H_16_O_6_, implying eight degrees of unsaturation, based on the adduct ion [M+Na]^+^ at *m/z* 315.0835 as determined from the ESI-(+)-HRMS data. The presence of an OH at position C-1' gave rise to the fragment ion at *m/z* 227.0315 attributed to the C_11_H_8_O_4_Na^+^ as observed in the HRMS/MS spectrum. In addition, the C_15_H_14_O_5_Na^+^ fragment ion was observed in the MS/MS spectrum of compound **1**, indicating the presence of two oxygen atoms in positions C-1' and C-2'.

The NMR spectra of compound **1** (Table 2) revealed the presence of a 7-methoxylated coumarin and in particular the occurrence of a 3-methylene-1,2,4-*n*-butanotriol chain in position 8 of the core coumarin structure. Specifically, in the ^1^H-NMR spectrum of **1**, two spin systems in the aromatic region were observed which were present in all isolated 8-substituted 7-methoxylated coumarins (compounds **1**–**5**, **7** and **8**). The spin system, corresponding to the aromatic protons H-5, H-6 consists of two doublets observed at δ 7.55 and 7.05, respectively, with an 8.8 Hz coupling constant. The respective aromatic carbons C-5 and C-6 resonate at δ 130.7 and 109.9, respectively (HMQC spectrum). The second spin system was comprised of two doublets at δ 6.25 (H-3) and 7.87 (H-4) with a coupling constant of 9.4 Hz typical for olefinic protons of an α-pyrone ring. Correlations of these protons with carbons at δ 113.6 and 146.5 were observed in the HSQC spectrum, leading to their characterization as C-3 and C-4, respectively. The methoxy group was deduced from the presence of a singlet integrating for three protons and resonating at δ 3.96. The carbon at δ 57.0 was attributed to the oxygenated carbon of the methoxy group by the cross peak correlation of this carbon with the methoxy protons detected in the HSQC spectrum. In addition, the quaternary carbons of the 7-methoxylated coumarin structure were revealed by the cross peaks detected in the HMBC spectrum.

**Table 2:** NMR Spectroscopic Data (600 MHz, CD_3_OH) for compounds **1**–**3**.

Position	1			2			3		
	δ_H_ (*J* in Hz)	δ_C_	HMBC	δ_H_ (*J* in Hz)	δ_C_	HMBC	δ_H_ (*J* in Hz)	δ_C_	HMBC
2		163.1			163.0			162.6	
3	6.25, d (9.4)	113.6	2, 9	6.29, d (9.4)	114.0	2, 9	6.26,d (9.4)	113.8	2, 9
4	7.87, d (9.4)	146.5	2, 3, 5, 10	7.95, d (9.4)	146.4	2, 5, 9, 10	7.90,d (9.4)	146.5	2, 5, 9, 10
5	7.55, d (8.8)	130.7	4, 7, 10	7.73, d (8.8)	132.2	4, 7, 10	7.63,d (8.8)	131.4	4, 7, 9, 10
6	7.05, d (8.8)	109.9	8, 9	7.17, d (8.8)	109.8	7, 8, 9	7.09,d (8.8)	109.9	7, 9, 10
7		162.4			162.3			162.9	
8		117.9			107.5			109.2	
9		114.8			115.0			114.8	
10		154.5			154.4			154.9	
1'	5.37, d (8.8)	70.6	7, 8, 10, 2'				5.52, d (5.9)	78.3	6, 7, 10, 2'
2'	4.94, d (8.8)	77.2	1', 3', 4', 5'		172.6		6.11, d (5.9/1.8)	78.2	3', 5'
3'		150.4			123.4			148.3	
4'a	4.08, dt (15.3/1.8)	62.8	3', 5'		165.1		4.74, dt (13.0/2.0)	72.5	8, 1', 3'
4'b	4.00, dt (15.3/1.8)		5'				4.62, m		8, 1', 3'
5'a	4.92, d (1.8)	112.0	2', 4'	6.18, brs	101.4		5.28, m	109.6	2', 3', 4'
5'b	4.91,d (1.8)		2', 4'						
1''								174.6	
2''							2.21, m	44.5	1'', 3'', 4'', 5''
3''							2.01, m	27.3	1'', 4'', 5''
4''							0.88, d (6.7)	22.7	2'', 3'', 5''
5''							0.89, d (6.7)	22.7	2'', 3'', 4''
CH_3_O	3.96, s	57.0	7	3.93, s	57.2	7	3.93, s	57.1	7
CH_3_				1.96, s	13.3	8, 2', 3', 4', 5'			

Specifically, the aromatic proton H-5 displayed correlations with the downfield shifted oxygenated quaternary carbons C-7 at δ 162.4 and C-10 at δ 154.5 as well as with the olefinic carbon C-4. Carbon C-7 was also correlated with the protons of the methoxy group. Moreover, the quaternary carbon at δ 114.8 was determined as C-9 based on its correlations with the aromatic proton H-6 and the olefinic proton H-3 while the carbon at δ 117.9 was assigned as C-8 due to its correlations with H-6. Finally, the carbonyl carbon at δ 163.1 was revealed from its cross peak correlation with the olefinic H-3.

The aliphatic chain at position 8 on the core structure was deduced as 3'-methylene-1',2',4' *n*-butanetriol based on certain resonances. Specifically, H-1' and H-2' were observed as doublets at δ 5.37 and 4.94, respectively, with an 8.8 Hz coupling constant, while the absence of a NOE cross peak between them suggested the *erythro* configuration of the hydroxyl groups. The C-1' and C-2' carbons were detected at δ 70.6 and 77.2 and correlated with the respective protons in the HSQC spectrum. The quaternary carbon C-3' resonated at 150.4 ppm as deduced from the HMBC spectrum. Methylene protons H-4'a and H-4'b were observed at δ 4.08 and 4.00 as two doublets of triplets with coupling constants of 15.3 and 1.8 Hz, while C-4' was resonated at δ 62.8. Finally, the protons at δ 4.92 and 4.91 were attributed to H-5' which correlated with the C-5' at δ 112.0 as observed in the HSQC spectrum. Finally, the position of the side chain was determined based on the correlation of H-1' (δ 5.37) with C-8 observed in the HMBC spectrum. Thus, compound **1** was identified as 8-(5-hydroxy-4-methyl-2-oxo-5*H*-fur-3-yl)-7-methoxy-2-chromenone to which the trivial name microfalcrin was given.

Compound **2** (peak 7, Rt 6.12 min) was isolated as a white amorphous powder presenting a characteristic UV spectrum (MeOH) with absorption maxima at 207 and 319 nm indicative of 7-oxygenated coumarin derivative. Its molecular formula was calculated as C_15_H_12_O_6_ from the adduct ion [M+Na]^+^ at *m/z* 311.0524 observed in the ESI-(+)-HRMS spectrum, implying eight degrees of unsaturation. The absence of characteristic fragment ion in MS/MS level is an indication of furan or lactone ring on the coumarin structure.

Like **1**, in the NMR spectra of **2** the spin systems corresponding to a typical 8-substituted 7-methoxylated coumarin were obvious. In addition to these characteristic resonances certain signals indicative to a butenolide (2-furanone) ring were observed (Table 2). Particularly, H-5' was detected as a broad singlet at δ 6.18 indicating the presence of a hydroxyl group on this carbon, and C-5' was observed at δ 101.4 in the HSQC spectrum. Furthermore, the protons of the methyl group in C-4' position resonated at δ 1.96 as a singlet and correlated with the upfield shifted carbon at δ 13.3 as observed in the HSQC spectrum. In order to determine the rest of the quaternary carbons of this ring and its position on the core structure, the HMBC spectrum was analyzed. Interestingly, the methyl protons displayed correlations with all the carbons of the butenolide ring (C-2', C-3', C-4', C-5') revealing the quaternary carbons C-2', C-3' and C-4' which resonated at δ 172.6, 123.4, and 165.1, respectively. In addition, another very useful correlation was observed between the same methyl protons and C-8 showing the position of the ring on the core structure. Hence, the 8-(5-hydroxy-4-methyl-2-oxo-5H-fur-3-yl)-7-methoxy-2-chromenone structure was deduced for compound **2** and the name microcoumaririn was given to it (Figure 2).

Compound **3** (peak 18, Rt 10.27 min) was isolated as a white amorphous powder. The UV spectrum in methanol presented absorption maxima at 206 and 320 indicating the presence of a 7-oxygenated coumarin. Its molecular formula was deduced as C_20_H_22_O_6_, suggesting 10 degrees of unsaturation, based on the [M+Na]^+^ adduct ion observed at *m/z* 381.1306 in the ESI-(+)-HRMS spectrum. Compound **3** presented a characteristic fragment ion at *m/z* 279.0631 corresponding to the neutral loss of an isovaleryl chain.

The NMR spectra of **3** were similar to the aforementioned compounds concerning the coumarin skeleton while additional signals revealed the presence of a 1',2',3',4'-tetrahydro-3'-methylene-1'-furanyl-2'-isovalerylester substitute (Table 2). In the ^1^H-NMR spectrum the H-1' of the tetrahydrofuran ring resonated at δ 5.52 as a doublet (*J* = 5.9 Hz) and H-2' was observed at δ 6.11 as doublet of doublets (*J* = 5.9 and 1.8 Hz). Based on the coupling constant value between H-1' and H-2', a *trans* configuration is assumed [8,18]. In the HSQC spectrum the respective carbons at δ 78.3 and 78.2 were determined and from their ppm values it could be deduced that both were oxygenated. The position of the ring at C-8 of the core structure was determined from the cross peak correlations of the H-1' with C-6 (^4^*J*), C-7 (^3^*J*), and C-10 (^3^*J*), observed in the HMBC spectrum. H-4'a was observed at δ 4.74 as a doublet of triplets (*J* = 13.0 and 2.0 Hz) and H-4'b at δ 4.62 as a multiplet; both H-4'a and H-4'b were correlated with a carbon at δ 72.5 as detected in the HSQC spectrum. H-5' resonated at δ 5.28 as a multiplet and C-5' was observed at δ 109.6 in the HSQC spectrum. Finally, the presence of the quaternary C-3 at δ 148.3 was revealed from its cross peak correlation with H-4'a and H-4'b and H-5' in the HMBC spectrum. The isovaleryl moiety was deduced from the presence of a carbon resonated at δ 174.6 attributed to the carbonyl C-1'' as observed from the cross peaks of H-2'' and H-3'' in the HMBC spectrum. H-2'' and H-3'' were detected as multiplets at δ 2.21 and 2.01, respectively, with their corresponding carbons resonating at δ 44.5 and 27.3. Finally, H-4'' and H-5'' were observed at δ 0.88 and 0.89 as two doublets integrating for six protons and both C-4'' and C-5'' resonated at δ 22.7 as observed in the HSQC spectrum. Thus, compound **3** was identified as 2-(7-methoxy-8-coumarinyl)-4-methylene-3,5-dihydro-2*H*-fur-3-yl 3-methylbutyrate and named micromelosidester (Figure 2).

### 2.3. Mass Spectrometry of 7-Methoxylated Coumarin Derivatives

The UPLC-HRMS & HRMS/MS profiling of the *M. falcatum* extracts as well as the analysis of the isolated 7-methoxylated coumarins by the direct infusion method revealed patterns that could be utilized for dereplication purposes (Figure 3). Specifically, even if both ionization modes were assessed only in positive mode stable precursor ions were observed in the full scan spectra. Moreover, all isolated compounds formed sodium adduct ions [M+Na]^+^ whereas the respective molecular or pseudomolecular ions were present in very low intensity or were even absent. Additionally, at the MS/MS level, diagnostic fragment ions were detected and attributed to specific structural features (Figures S12–S32).

For instance, the presence of a free OH group at the C-2' position of 8-substituted 7-methoxylated coumarins seems to favor the cleavage of the C-1'/C-2' bond and the fission of the side chain generating stable C_11_H_8_O_4_Na^+^ ions observed at *m/z* 227.0317. This diagnostic ion was observed only in the HRMS/MS spectra of isolated compounds **1** and **7** as well as in the HRMS/MS spectra of peaks 1, 2, 5, 14 (Table 1). Another characteristic fragment ion was observed at *m/z* 297.0736 in the MS/MS spectra and attributed to the C_15_H_14_O_5_Na^+^ ions. It seems that coumarins with oxygenated C-1' and C-2' generate this fragment ion through the loss of H_2_O in a certain stage of their fragmentation. Only compounds **1** and **7** from isolated ones and peaks 1–3, 5, 9, 12–15 revealed this fragment ion as confirmed by the MS/MS spectra. It seems that the presence of 3'-methylen-1',2',4'-*n*-butanotriol side chain is evident only when both fragment ions are present (**1** and **7**). Moreover, compounds possessing an isovaleryl side chain (**3**, **7**, **8**) presented a characteristic loss of 102 amu corresponding to the cleavage of the ester bond and the elimination of this chain. Independently of the substitute structure (chain or ring) the ester bond seems to be fragile even under the mild CID conditions. This loss was also observed in the HRMS/MS spectra of peaks 12–14 and 16–19 suggesting that these compounds incorporate an isovaleryl side chain in their structures (Table 1).

**Figure 3:**
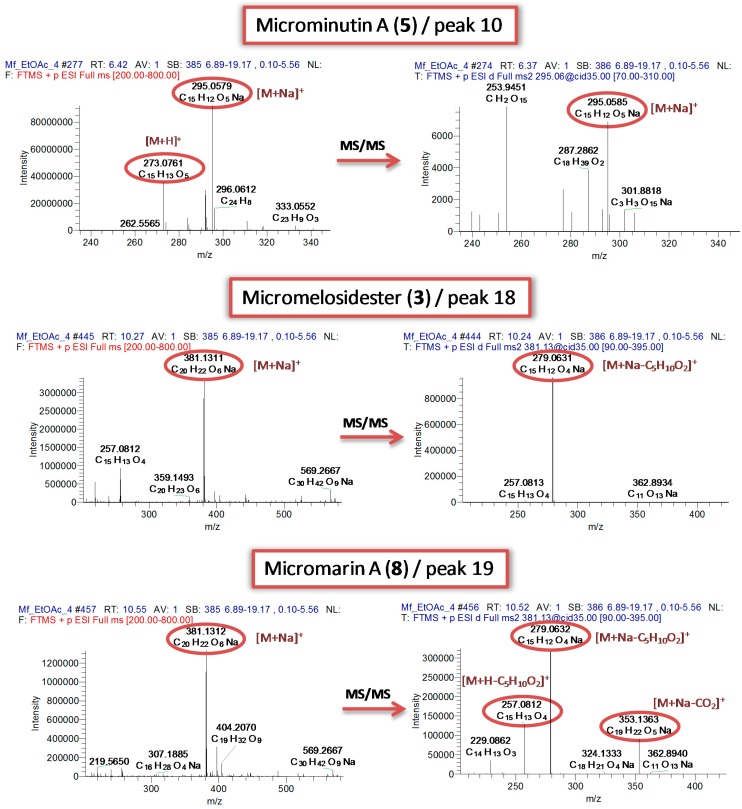
Characteristic HRMS and HRMS/MS spectra of representative 7-methoxylated coumarins.

Interestingly, the presence of an additional tetrahydrofuran or 2-furanone ring at position C-6 or C-8 lead to the formation of stable molecular adduct ions [M+Na]^+^ which did not give any fragment ion in MS/MS level in any CID conditions and this was observed in compounds **2**, **4**–**6** and peaks 4, 7, 10, 11. Thus, the absence of fragment ions could be indicative for the presence of any abovementioned ring on the basic 7-methoxylated 8-substituted coumarin structure.

Summarizing these observations, the developed method enabled the detection of 8-substituted 7-methoxylated coumarins, the discrimination of coumarins with open side chains *vs.* additional rings at C-8 as well as the identification of esterified derivatives. At this point, it is important to note that the proposed structures were verified after isolation and structure elucidation by NMR validating our approach. Finally, it is worth noting that LTQ-Orbitrap platform displayed excellent robustness and high reproducibility between LC-MS^n^ and direct infusion spectra confirming this statement from previous studies [19].

### 2.4. Anti-Inflammatory Evaluation of M. falcatum Extracts and Representative Compounds

In order to estimate the anti-inflammatory activity of *M. falcatum*, the EtOAc extract as well as two representative coumarin derivatives were evaluated for the inhibition of the pro-inflammatory mediator NF-κB induction and NO production. **5** and **7**, representative of the presence of an additional ring and free side chain, respectively, were selected to be screened. NF-κB plays a critical role in the inflammation process inducing the transcription of pro-inflammatory mediators such as inducible nitric oxide synthase (iNOS), cyclooxygenase 2 (COX-2), TNF-α, interleukin (IL)-1β and IL-6 [20]. Interestingly, significant activity was found in the EtOAc extract which showed 46% inhibition of NO production at 20 µg/mL (Figure S33). However, this extract did not show any effect on NF-κB in a luciferase reporter assay (Figure S34). Unluckily, compounds **5** and **7**, the major compounds found in almost all extracts did not present any inhibition, indicating that the observed activity of the extract must be due to minor compounds and/or to synergy effects.

## 3. Experimental Section

### 3.1. General Procedures

UV spectra were obtained using spectroscopic grade MeOH on a Shimadzu-160A spectrophotometer (Shimadzu, Kyoto, Japan). All nuclear magnetic resonance (NMR) spectra were recorded on a Bruker Avance III 600 spectrometer (Bruker BioSpin, Rheinstetten, Germany) operating at 600 and 150 MHz for ^1^H and ^13^C, respectively, equipped with a 5 mm BBI probe and by using CD_3_OD (Sigma-Aldrich, Steinheim, Germany) as deuterated solvent. Chemical shifts (δ) are expressed in ppm with reference to the solvent signals (δ H 3.34/δ C 49.86) and coupling constants in Hz. The 2D NMR experiments (COSY, HSQC, HMBC, and NOESY) were performed using standard Bruker microprograms. HPLC-DAD semi preparative isolation was performed on a Thermo Finnigan apparatus (Bremen, Germany) equipped with a PDA Spectra System UV6000LP. For the separation an RP-18 Discovery semi-preparative column, 250 mm × 10 mm i.d.; 5 μm, (Supelco, SigmaAldrich, Steinheim, Germany) was used. MS grade acetonitrile (ACN), water (H_2_O) and formic acid (FA) were purchased from Sigma-Aldrich (Darmstadt, Germany).

### 3.2. Plant Material

The leaves of *M. falcatum* (Lour.) Tan. were collected in Kampot of Cambodia at an altitude of 41 m, in March 2006. The collection and identification of the plant material was performed by Professor Sun Kaing Cheng and Dr. Sovannmoly Hul and a voucher specimen was deposited at the Herbarium of the National Museum of Natural History in Paris, France.

### 3.3. Extraction of M. falcatum

Initially, 10 g of dried and pulverized leaves of *M. falcatum* were extracted successively with DCM, MeOH and H_2_O (3 × 150 mL for each solvent) for two hours in an ultrasonic bath. The extracts were concentrated under reduced pressure to obtain DCM (321.5 mg), MeOH (400.3 mg) and H_2_O (1538.7 mg) extracts. Another protocol, focusing on the targeted isolation of the alkaloids was also applied for the extraction of 10 g of dried and pulverized leaves of *M. falcatum*. The plant material was initially extracted with EtOAc (3 × 150 mL) in an ultrasonic bath for 2 h. The extracts obtained were combined and evaporated under reduced pressure. A total of 244.4 mg of EtOAc extract was obtained. Thereafter, the plant material was alkalinized with 10% of NH_4_OH (60 mL) and extracted successively with EtOAc (150 mL) and MeOH (150 mL) for 2 h in an ultrasonic bath. The extracts obtained after alkalinization were partitioned with 10% HCl until the negative reaction of the aqueous phase using Mayer’s reagent, then alkalinized to pH 8–9 with 28% NH_4_OH and extracted with DCM using Mayer’s reagent to control the process. Finally, an organic fraction (EtOAc_A) (40.2 mg) and an aqueous fraction (EtOAc_B) (14.2 mg) of the EtOAc extract as well as an organic fraction (MeOH_A) (50.2 mg) and an aqueous fraction (MeOH_B) (121.2 mg) of the MeOH extract were obtained.

### 3.4. Sample Preparation

All the extracts were diluted in a mixture of ACN/H_2_O (1:1) to obtain a 500 μg/mL solution (stock solution). Then, final solutions of 100 μg/mL were prepared by diluting 200 μL of the stock solution in 800 μL of H_2_O. Isolated compounds were infused as 10 μg/mL solutions prepared in ACN.

### 3.5. HPLC-DAD Analysis

The profiling of the *M. falcatum* extracts was initially carried out using a HPLC-DAD Thermo Spectra System apparatus (Thermo Scientific, Bremen, Germany). The same HPLC system was applied for profiling and isolation purposes. Concerning the profiling, the analysis was performed using a RP-18 HS C18 Discovery column (250 mm × 4.6 mm i.d.; 5 μm, Supelco, SigmaAldrich, Steinheim, Germany). The mobile phase consisted of ACN and water with 0.1% formic acid and the flow rate was set at 1 mL/min. The elution program was composed of an initial gradient elution step of 60 min from 5% ACN to 100% ACN followed by 5 min of isocratic step (100% ACN). A step of 5 min returning to the initial condition and finally 5 min of equilibration were followed. The detection was performed using a UV absorption range of 200–600 nm and three different UV channels at 254, 280 and 365 nm were chosen to monitor the run. The system control and the analysis of the data were performed using Chromquest 4.2 software.

### 3.6. Mass Spectrometry

The profiling of *M. falcatum* extracts was carried out using a LTQ-Orbitrap apparatus (Thermo Finnigan, San Jose, CA, USA). The UPLC system is equipped with an Acquity pump, autosampler (10 μL sample loop, partial loop injection mode, 3 μL injection volume). A fast gradient method of 20 min was developed for the analysis on a BEH C_8_ (100 mm × 2.1 mm i.d.; 1.7 μm) Acquity column. The mobile phase consisted of solvent A (0.1% formic acid in H_2_O) and solvent B (ACN) and the flow rate was set at 250 μL/min. The elution was performed isocratically for 1 min with 5% solvent B, then a linear gradient up to 95% of B for 15 min was succeeded; an isocratic step of 1 min was followed with 95% of B returning to the initial conditions (5% solvent B) in 1 min and concluding with a 3 min conditioning step of 5% solvent B. For these experiments, the ESI source in positive mode was used and the parameters of the operation were set as follows: spray voltage at 3.5 V, capillary temperature at 350 °C, capillary voltage at 35 V and tube lens at 80 V. Flow rate of nitrogen was used as sheath and auxiliary gas, and was adjusted to 45 and 10 arb units, respectively. Full scans were acquired in profile mode at 30000 (FWHM) resolution and a mass range of *m/z* 200–800. In parallel, a data dependent scan was acquired applying collision energy (CID) at 35% and dynamic exclusion enabled. An activation time of 30 ms and a window of 2 u were set to isolate the precursor ions. For direct infusion experiments, a syringe pump (500 μL syringe, Hamilton Bonaduz AG, Bodanuz, Switzerland) was coupled with the LTQ-Orbitrap mass spectrometer. For these experiments, the ESI operation parameters and the acquisition parameters were identical to the aforementioned apart from the nitrogen flow which was adjusted to 12 and 6 arb units as sheath and auxiliary gas, respectively. The flow rate of the injection was set at 5 μL/min. Regarding the MS/MS experiments, the collision energy (CID) was adjusted between 20% and 35% based on the requirements of each compound under analysis. The activation time was set at 30 ms (q, 0.25) and a window of 2 u was used to isolate the precursor ions. The acquisition and the processing of the data were performed using Xcalibur software (version 2.0.7, Thermo Scientific).

### 3.7. Targeted Isolation of 7-Oxygenated Coumarins

After the screening of all obtained extracts by HPLC-PDA and UPLC-ESI(+)-HRMS, the DCM and EtOAc extracts and the organic fraction of the EtOAc extract after the alkalization (EtOAc_A) were selected for the isolation of the coumarin derivatives using semi-preparative HPLC-PDA. A universal elution method was used for the isolation of coumarins from all the aforementioned extracts. The elution program consisted of ACN and H_2_O as mobile phase, a flow rate of 3 mL/min and injections corresponding to 3 mg of plant extract on column were performed. A linear gradient was applied for 60 min starting from 95% to 100% ACN then followed by 5 min of isocratic elution with 100% ACN, a step of 5 min to return to the initial conditions and finally 5 min of re-equilibration. From the DCM extract, microfacrin (**1**, 0.6 mg), microcoumaririn (**2**, 0.6 mg), micromeloside A (**4**, 0.8 mg), microminutin (**5**, 11.7 mg), micromelin (**6**, 1.0 mg), micromarin B (**7**, 1.1 mg) and micromarin A (**8**, 3.6 mg) were afforded. The purification of the EtOAc extract by semi-preparative HPLC-PDA yielded microfacrin (**1**, 0.4 mg), microcoumaririn (**2**, 0.6 mg) microminutin (**5**, 15.6 mg), micromelin (**6**, 1.6 mg), micromarin B (**7**, 2.1 mg) and micromarin A (**8**, 2.0 mg). Finally from the EtOAc_A fraction microcoumaririn (**2**, 0.7 mg), micromelosidester (**3**, 1.6 mg) and microminutin (**5**, 1.4 mg) were isolated. Unfortunately, the low quantity of the purified compounds did not allow further investigation for the determination of absolute configuration of the derivatives with stereocenters.

*Microfalcrin* (**1**): white amorphous powder; UV (MeOH) λ_max_ (logε) 206 (3.68), 319 (3.10); ^1^H- and ^13^C-NMR data, see Table 2; Positive ESI-HRMS *m/z* (rel.int.): 315.0835 [M+Na]^+^ (100) (calcd for C_15_H_16_O_6_Na, 315.0839).

*Microcoumaririn* (**2**): white amorphous powder; UV (MeOH) λ_max_ (logε) 207 (3.52), 319 (2.99); ^1^H- and ^13^C-NMR data, see Table 2; Positive ESI-HRMS *m/z* (rel.int.): 311.0524 [M+Na]^+^ (100) (calcd for C_15_H_12_O_6_Na, 311.0526), 289.0715 [M+H]^+^ (10).

*Micromelosidester* (**3**): white amorphous crystal; UV (MeOH) λ_max_ (logε) 206 (4.07), 320 (3.60); ^1^H- and ^13^C-NMR data, see Table 2; Positive ESI-HRMS *m/z* (rel.int.): 381.1306 [M+Na]^+^ (100) (calcd for C_20_H_22_O_6_Na, 381.1308), 359.1495 [M+H]^+^ (10).

### 3.8. Measurement of Nitric Oxide Production in LPS-Induced RAW264.7 Cells

RAW 264.7 cells (1 × 10^5^ cells/well) were plated in 96-well plates for 24 h at 37 °C, 5% CO_2_. The cells were then treated with test samples (20 µg/mL) for 30 min, followed by induction with LPS (1 µg/mL) for an additional 20 h. Resveratrol (20 µg/mL) was used as positive control. An equal volume of media containing the released nitrite and Griess reagent were mixed and absorbance was measured at 540 nm. Results are expressed as a percentage relative to control (LPS-induced) samples.

## 4. Conclusions

The HPLC-DAD and UPLC-ESI(+)-HRMS based profiling of different *M. falcatum* extracts was proven a useful tool for the targeted isolation of coumarins and their dereplication in complex mixtures. Specifically, following a certain dereplication strategy 19 in total, 7-oxygenated coumarins were detected, eight coumarins were isolated and unambiguously identified by 1 and 2D NMR and three were proven to be new natural products, namely microfalcrin, microcoumaririn and micromelosidester. Further investigation of these compounds by HRMS and HRMS/MS revealed specific spectrometric patterns that could be utilized for the rapid detection and characterization of 7-methoxylated coumarin derivatives. Finally, the traditional use of this medicinal plant may be supported by the significant inhibition of NO production observed in the EtOAc extract while in contrast the representative coumarin derivatives assayed failed to display significant activity.

## Supplementary Materials

Supplementary materials can be accessed at: http://www.mdpi.com/1420-3049/19/9/15042/s1.

## Supplementary Files

Supplementary File 1
