# Supplementary material for: Rapid Identification of Coumarins from Micromelum falcatum by UPLC-HRMS/MS and Targeted Isolation of Three New Derivatives
Source: Molecules. 2014 Sep 19;19(9):15042–57. doi: 10.3390/molecules190915042 (PMC6271814; doi:10.3390/molecules190915042)

Supplementary Materials

Figure S1. HPLC-DAD chromatograms of all obtained *Miromelum* extracts in 280 nm
Figure S2. HPLC-DAD chromatograms of EtOAc, EtOAc_A and EtOAc_B extracts in 280 nm
Figure S3. ^{1}H-NMR spectrum of microfalcrin (**1**) in CD_3OD
Figure S4. HSQC spectrum of microfalcrin (**1**) in CD_3OD
Figure S5. HMBC spectrum of microfalcrin (**1**) in CD_3OD
Figure S6. ^{1}H-NMR spectrum of microcoumaririn (**2**) in CD_3OD
Figure S7. HSQC spectrum of microcoumaririn (**2**) in CD_3OD
Figure S8. HMBC spectrum of microcoumaririn (**2**) in CD_3OD
Figure S9. ^{1}H-NMR spectrum of micromelosidester (**3**) in CD_3OD
Figure S10. HSQC spectrum of micromelosidester (**3**) in CD_3OD
Figure S11. HMBC spectrum of micromelosidester (**3**) in CD_3OD
Figure S12. Full scan MS and MS/MS spectra of peak 1 of Table 1
Figure S13. Full scan MS and MS/MS spectra of peak 3 of Table 1
Figure S14. Full scan MS spectrum of peak 4 of Table 1
Figure S15. Full scan MS and MS/MS spectra of peak 5 of Table 1
Figure S16. Full scan MS and MS/MS spectra of peak 6 of Table 1
Figure S17. Full scan MS spectrum of peak 7 of Table 1
Figure S18. Full scan MS and MS/MS spectra of peak 8 of Table 1
Figure S19. Full scan MS and MS/MS spectra of peak 9 of Table 1
Figure S20. Full scan MS and MS/MS spectra of peak 12 of Table 1
Figure S21. Full scan MS and MS/MS spectra of peak 14 of Table 1
Figure S22. Full scan MS and MS/MS spectra of peak 15 of Table 1
Figure S23. Full scan MS and MS/MS spectra of peak 16 of Table 1
Figure S24. Full scan MS and MS/MS spectra of peak 17 of Table 1
Figure S25. Full scan MS and MS/MS spectra of microfalcrin (**1**) in ESI(+)
Figure S26. Full scan MS spectrum of microcoumaririn (**2**) in ESI(+)
Figure S27. Full scan MS and MS/MS spectra of micromelosidester (**3**) in ESI(+)
Figure S28. Full scan MS spectrum of micromeloside A (**4**) in ESI(+)
Figure S29. Full scan MS spectrum of microminutin (**5**) in ESI(+)
Figure S30. Full scan MS spectrum of micromelin (**6**) in ESI(+)
Figure S31. Full scan MS and MS/MS spectra of micromarin B (**7**) in ESI(+)
Figure S32. Full scan MS and MS/MS spectra of micromarin A (**8**) in ESI(+)
Figure S33. Percentage of nitrite oxide production relative to control (LPS-induced); Resv: Resveratrol, Mmin: Microminutin (**5**), Mmar B: Micromarin B (**7**); MmEtOAc: EtOAc extract
Figure S34. Percentage of NFκB induction relative to control (TNF-α treated); Resv: Resveratrol, Mmin: Microminutin (**5**), Mmar B: Micromarin B (**7**); MmEtOAc: EtOAc extract

Figure S1. HPLC-DAD chromatograms of all obtained *Miromelum* extracts in 280 nm.

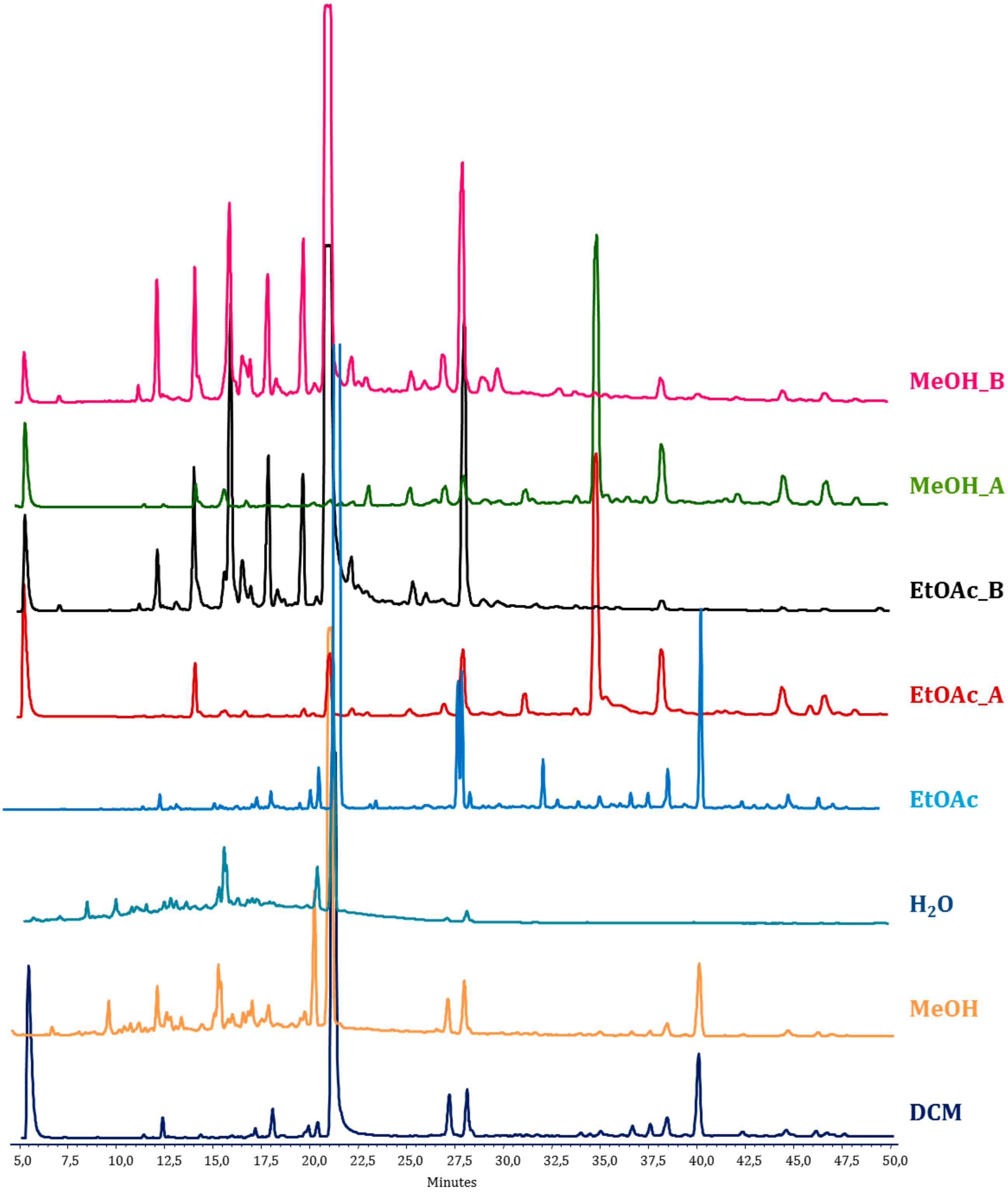

Figure S2. HPLC-DAD chromatograms of EtOAc, EtOAc_A and EtOAc_B extracts in 280 nm.

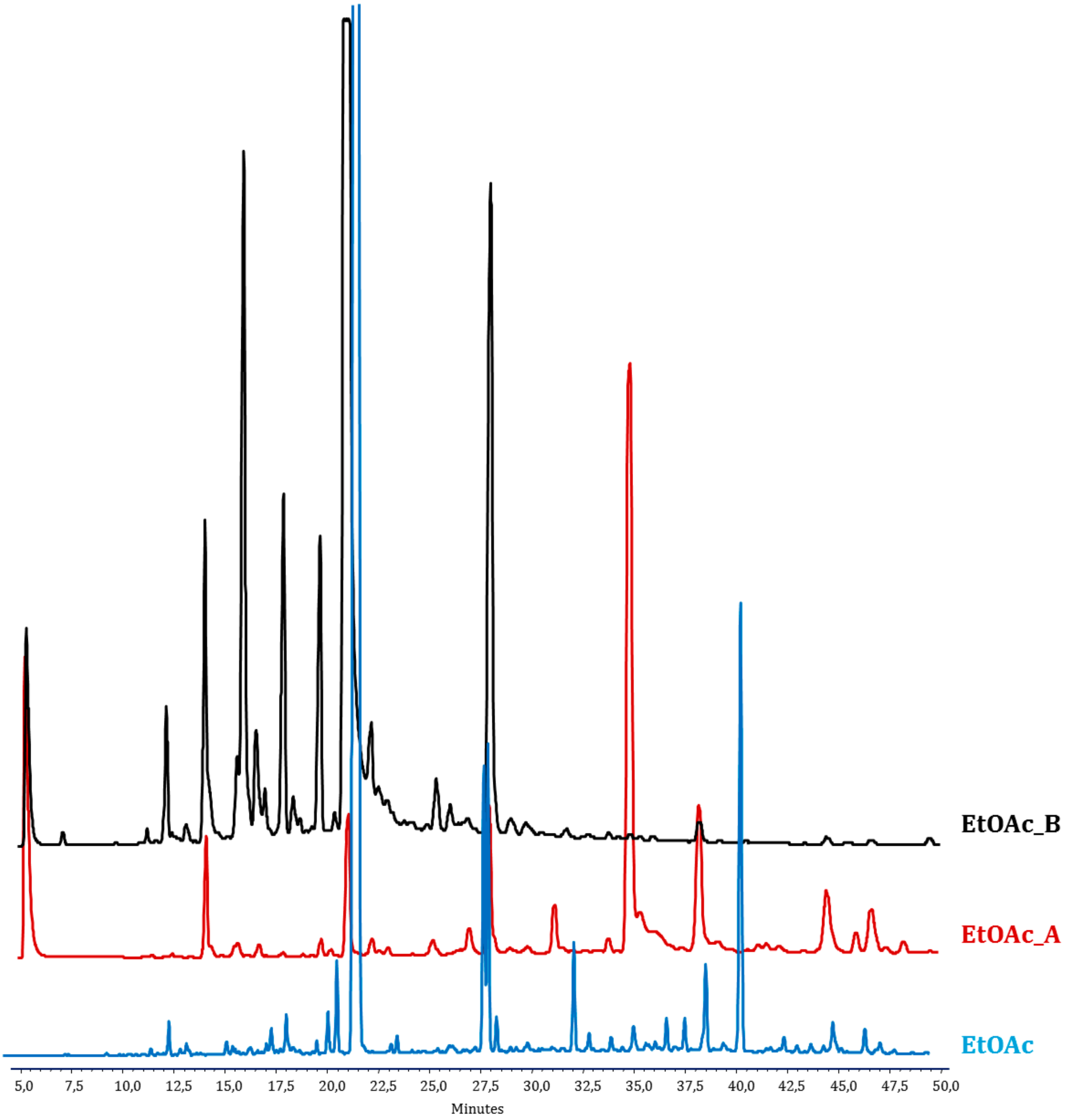

Figure S3. ^{1}H-NMR spectrum of microfalcrin (**1**) in CD_3OD.

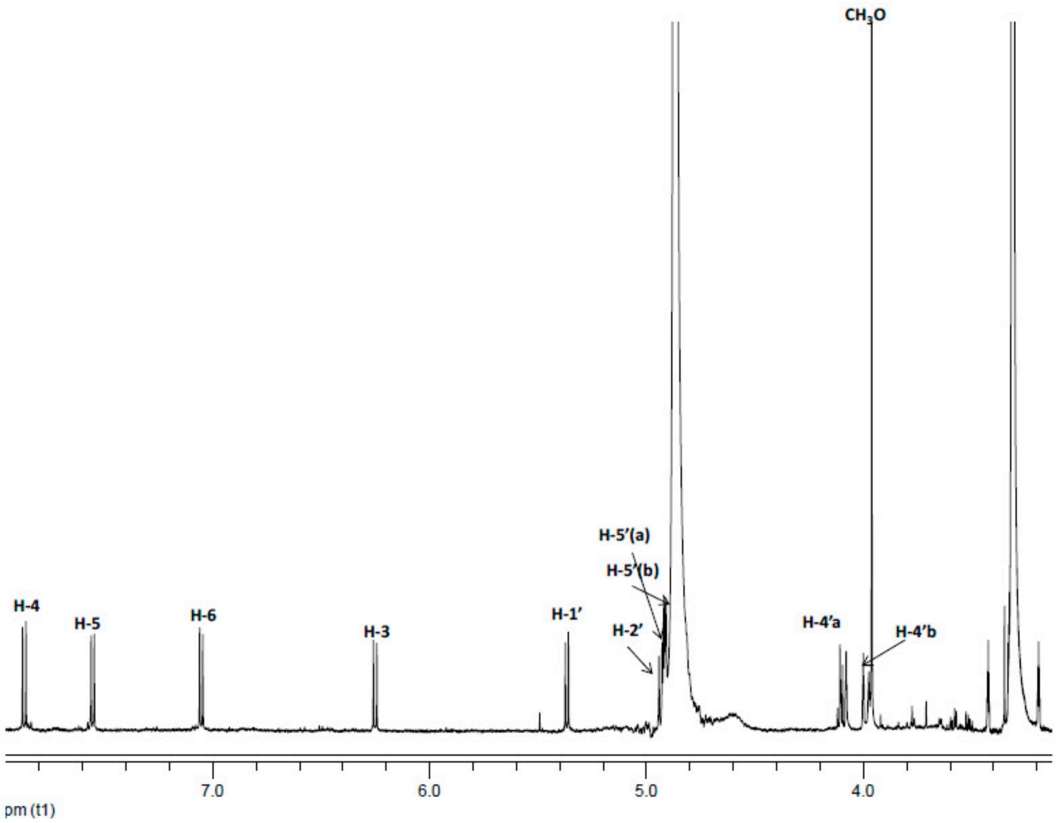

Figure S4. HSQC spectrum of microfalcrin (**1**) in CD_3OD.

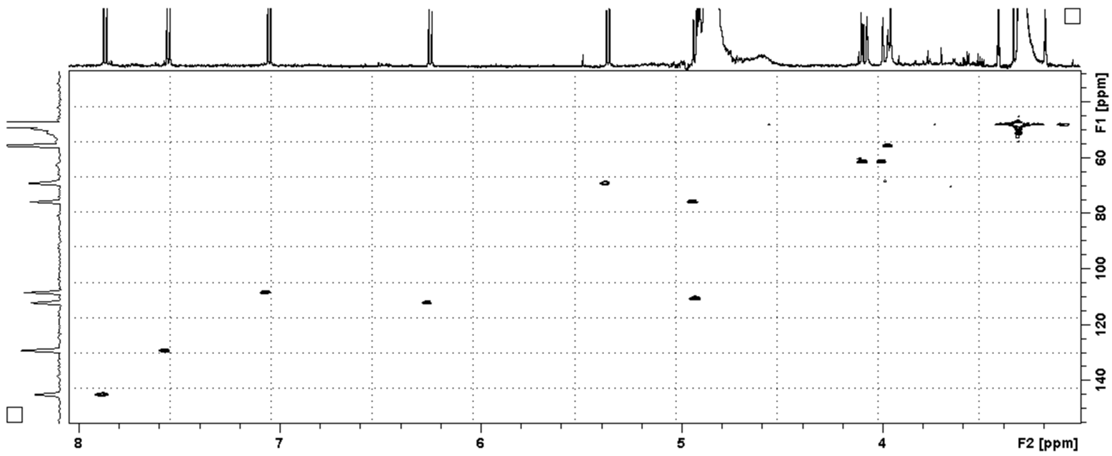

Figure S5. HMBC spectrum of microfalcrin (**1**) in CD_3OD.

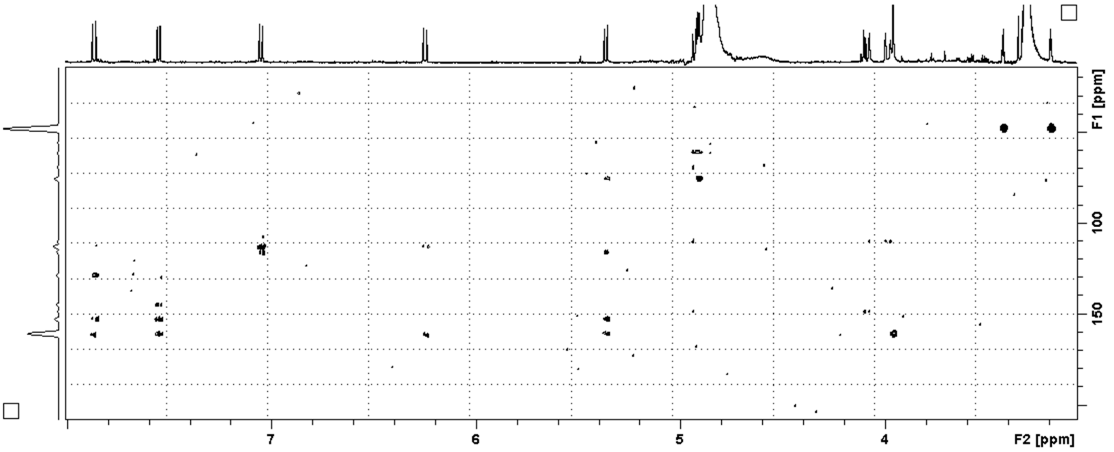

Figure S6. ^{1}H-NMR spectrum of microcoumaririn (**2**) in CD_3OD.

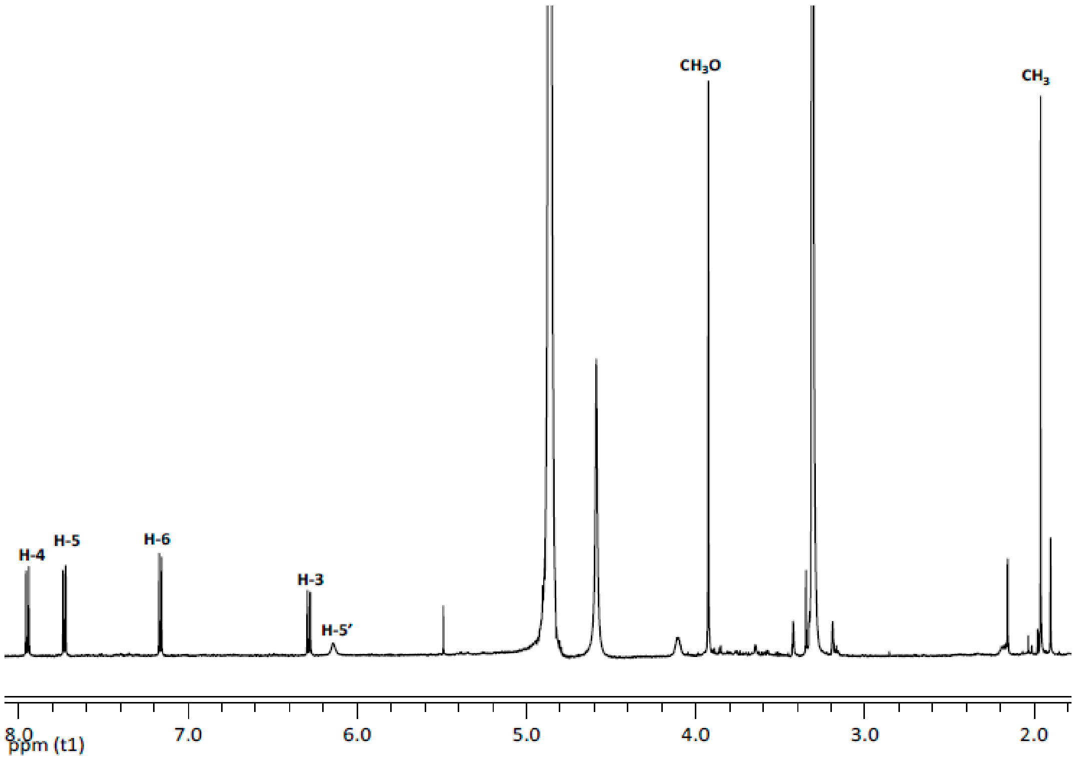

Figure S7. HSQC spectrum of microcoumaririn (**2**) in CD_3OD.

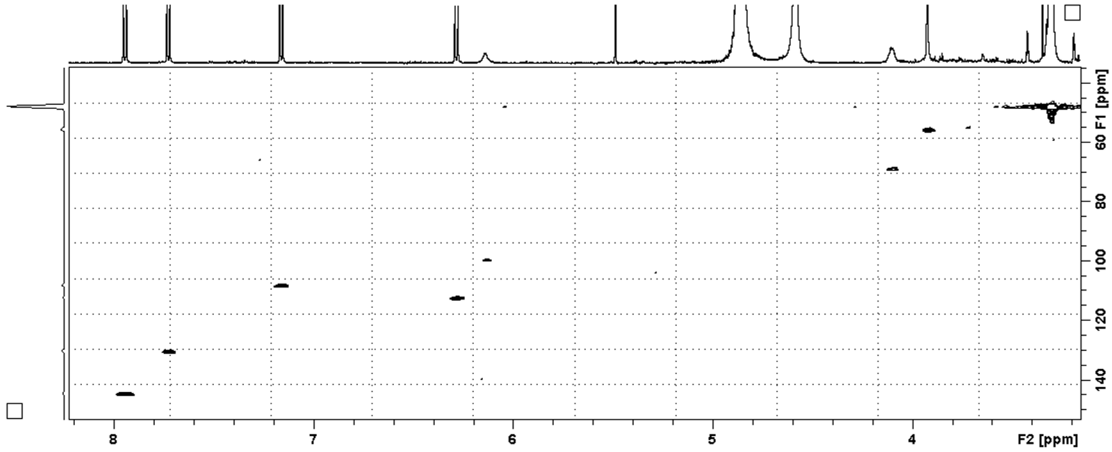

Figure S8. HMBC spectrum of microcoumaririn (**2**) in CD_3OD.

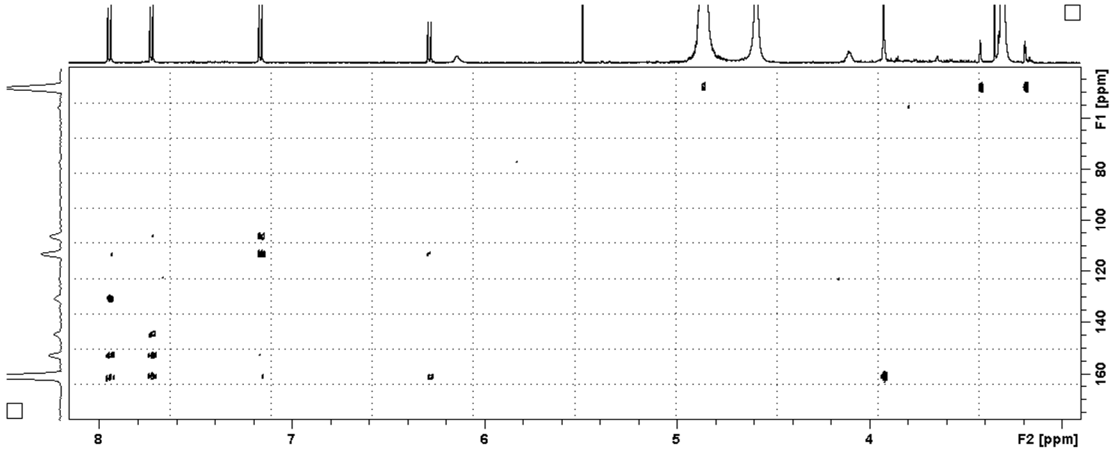

Figure S9. ^{1}H-NMR spectrum of micromelosidester (**3**) in CD_3OD.

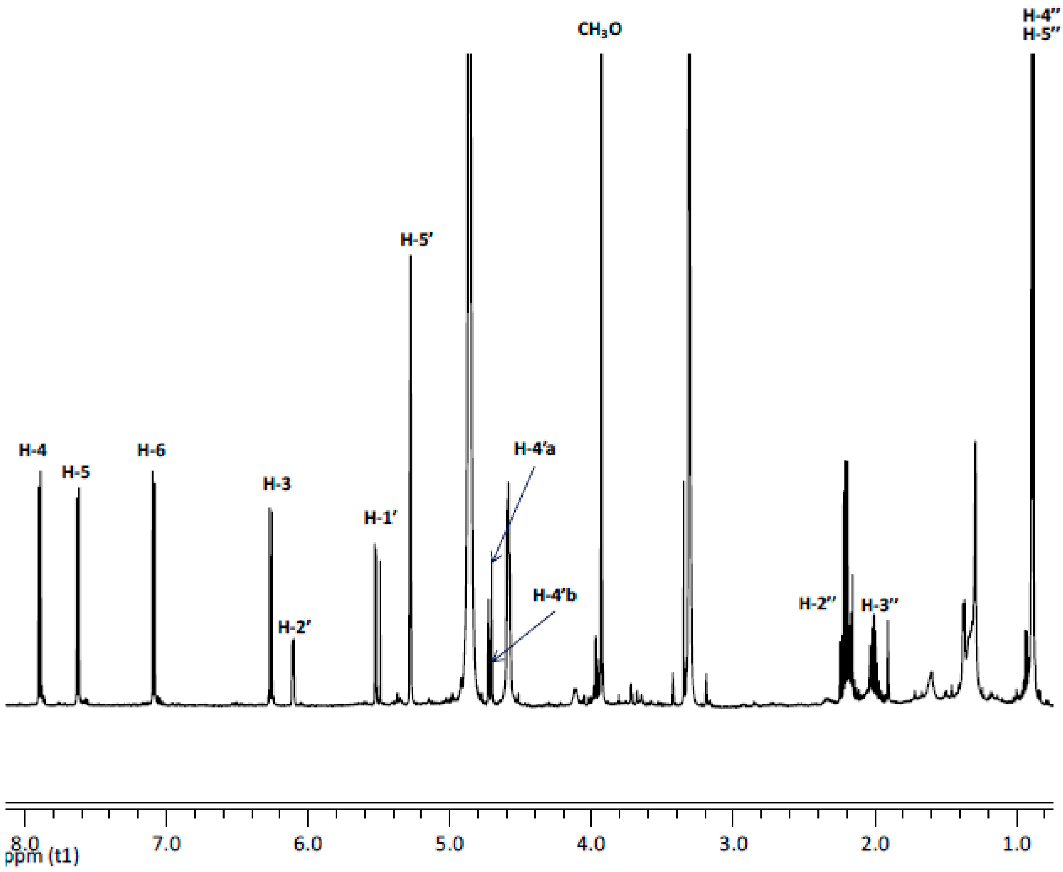

Figure S10. HSQC spectrum of micromelosidester (**3**) in CD_3OD.

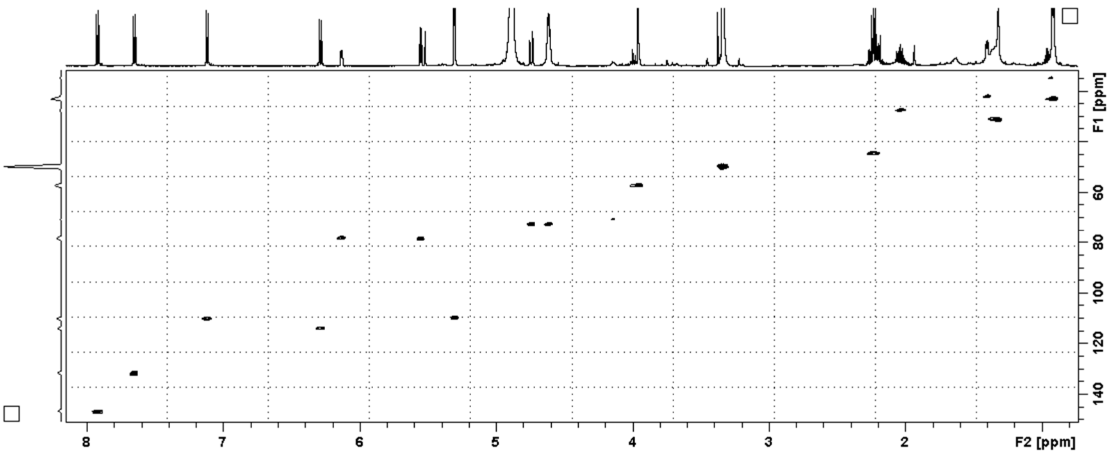

Figure S11. HMBC spectrum of micromelosidester (**3**) in CD3OD.

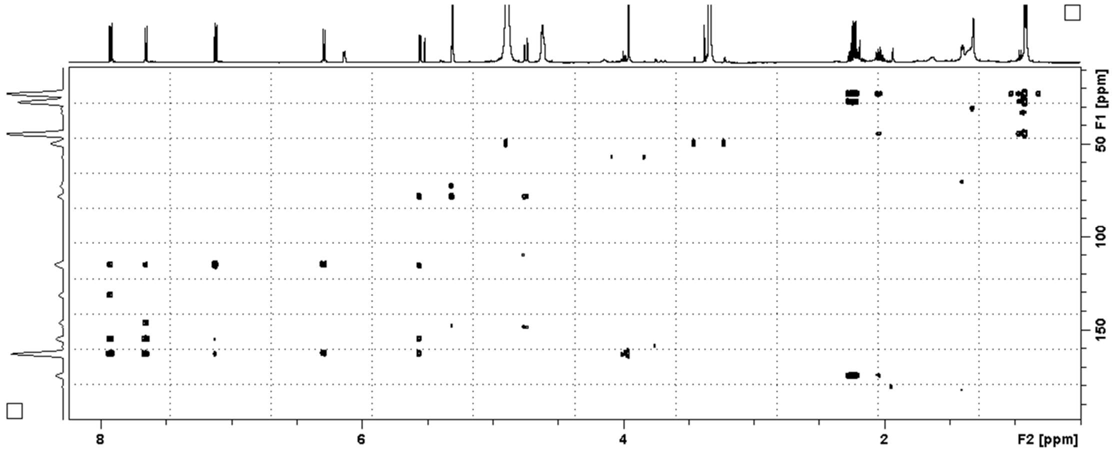

Figure S12. Full scan MS (**A**) and MS/MS (**B**) spectra of peak 1 of Table 1.

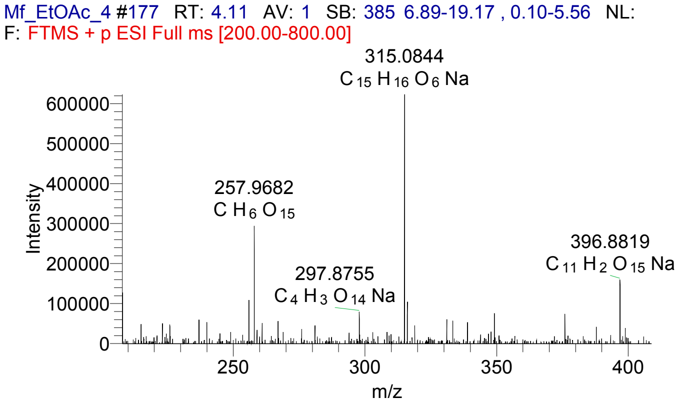

(**A**)

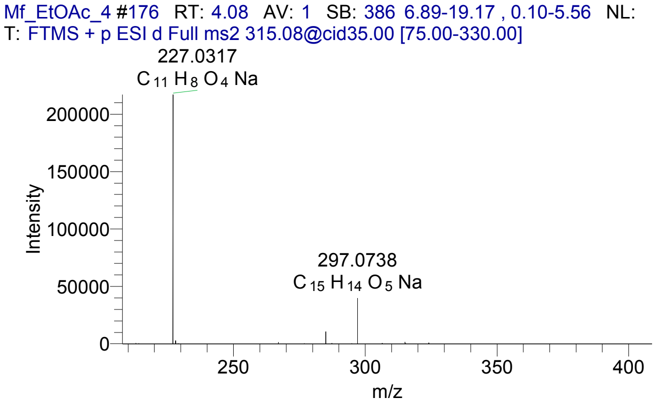

(**B**)

Figure S13. Full scan MS (**A**) and MS/MS (**B**) spectra of peak 3 of Table 1.

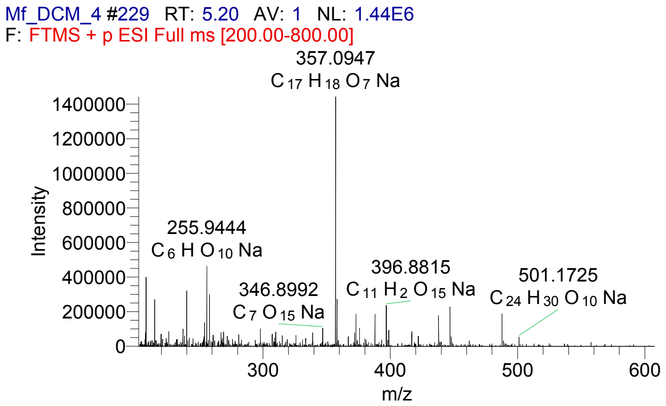

(**A**)

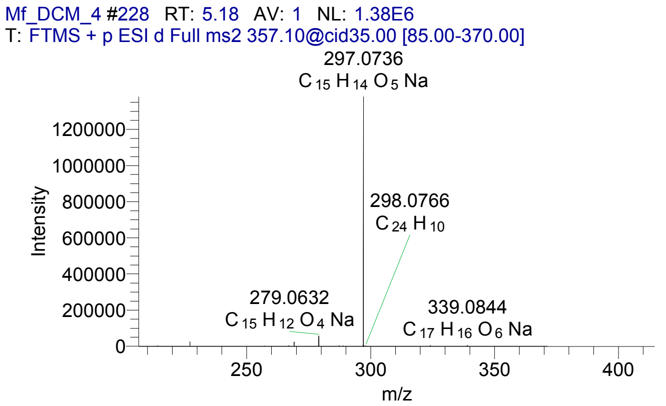

(**B**)

Figure S14. Full scan MS spectrum of peak 4 of Table 1.

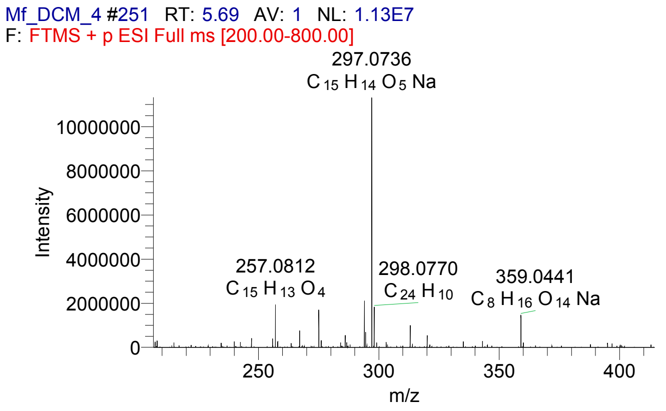

Figure S15. Full scan MS (**A**) and MS/MS (**B**) spectra of peak 5 of Table 1.

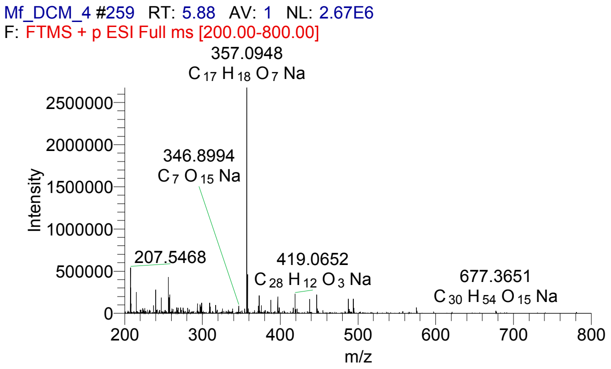

(**A**)

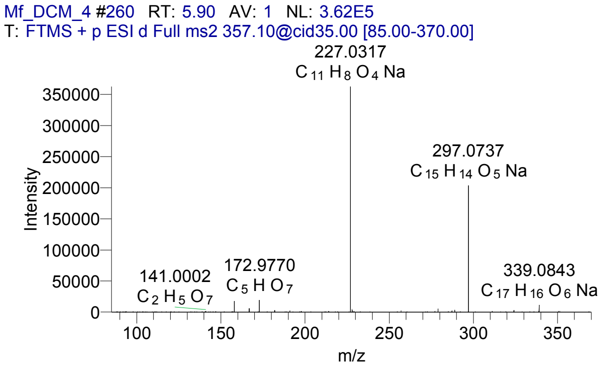

(**B**)

Figure S16. Full scan MS (**A**) and MS/MS (**B**) spectra of peak 6 of Table 1.

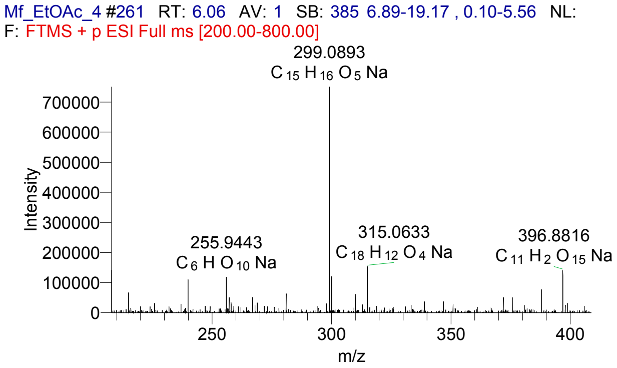

(**A**)

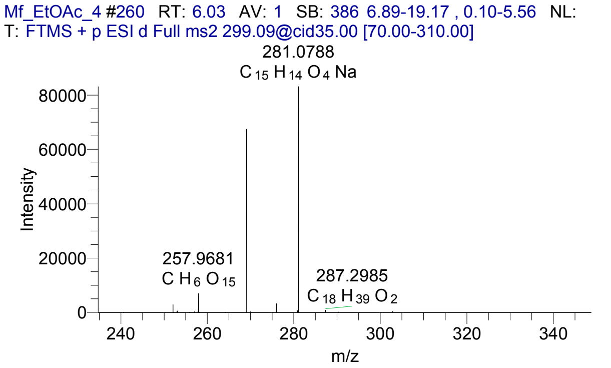

(**B**)

Figure S17. Full scan MS spectrum of peak 7 of Table 1.

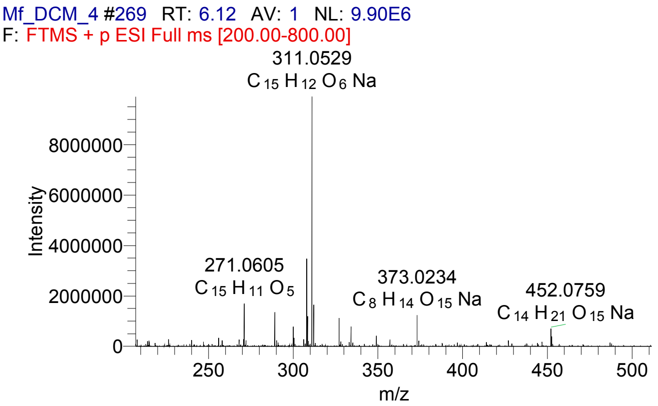

Figure S18. Full scan MS (**A**) and MS/MS (**B**) spectra of peak 8 of Table 1.

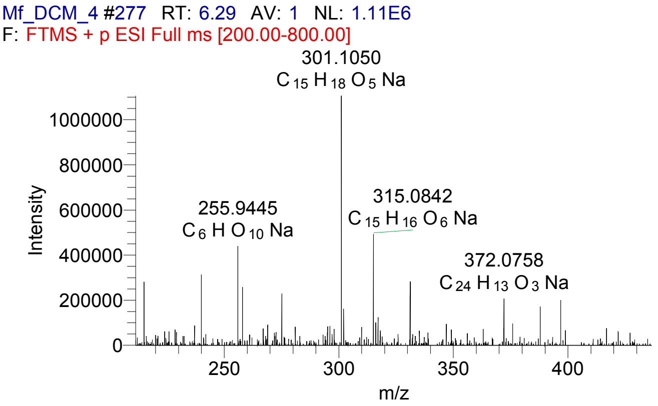

(**A**)

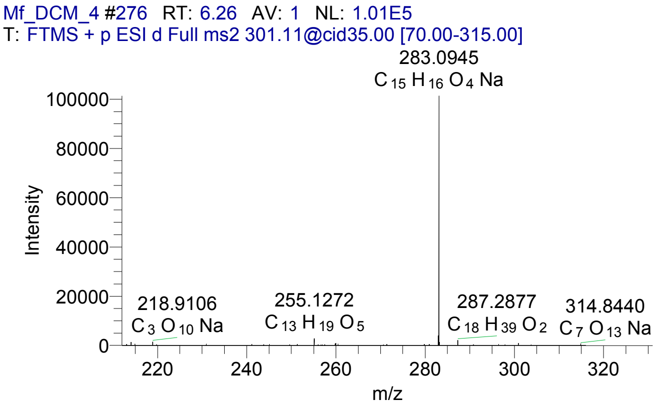

(**B**)

Figure S19. Full scan MS (**A**) and MS/MS (**B**) spectra of peak 9 of Table 1.

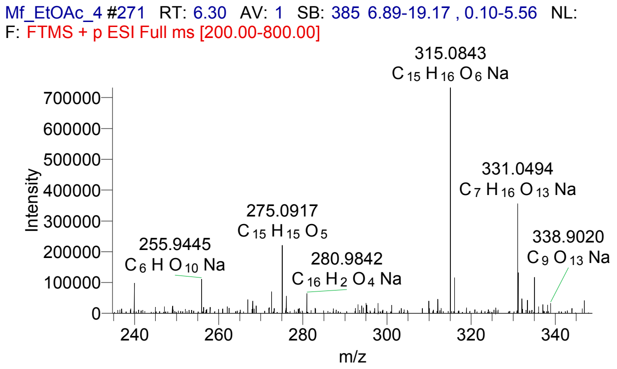

(**A**)

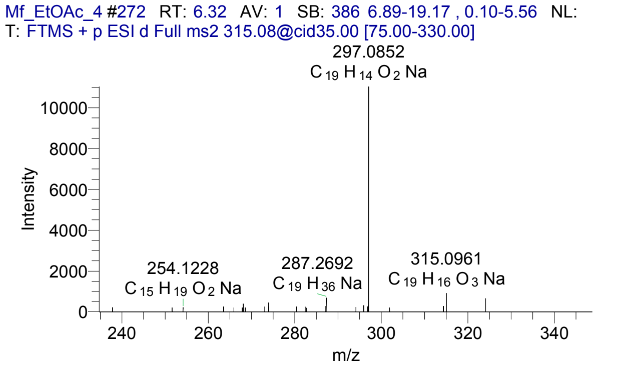

(**B**)

Figure S20. Full scan MS (**A**) and MS/MS (**B**) spectra of peak 12 of Table 1.

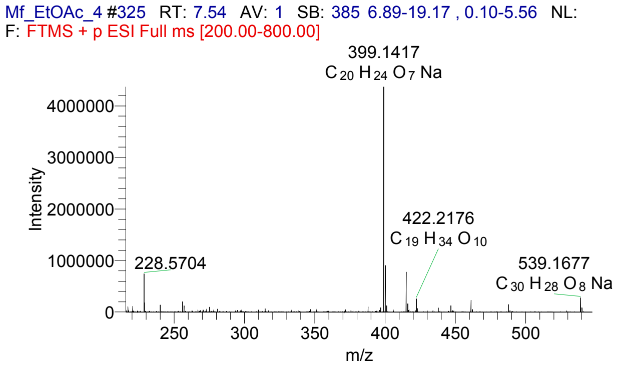

(**A**)

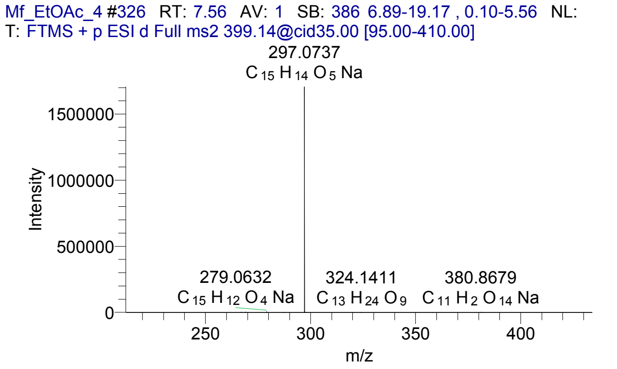

(**B**)

Figure S21. Full scan MS (**A**) and MS/MS (**B**) spectra of peak 14 of Table 1.

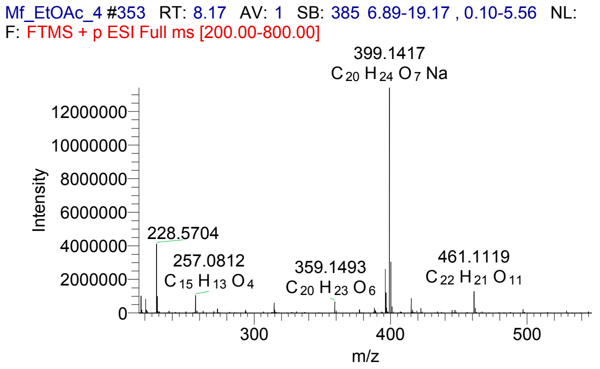

(**A**)

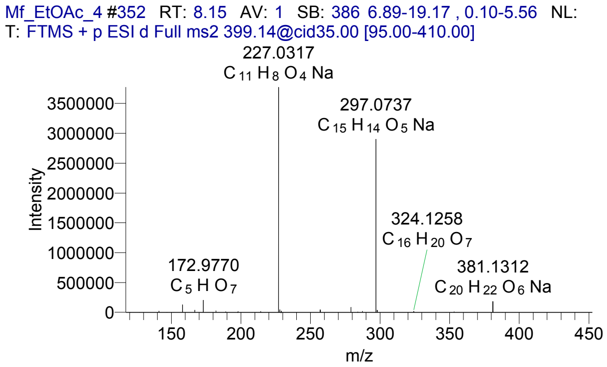

(**B**)

Figure S22. Full scan MS (**A**) and MS/MS (**B**) spectra of peak 15 of Table 1.

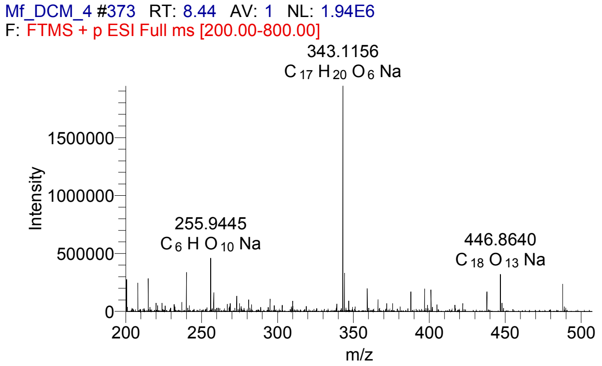

(**A**)

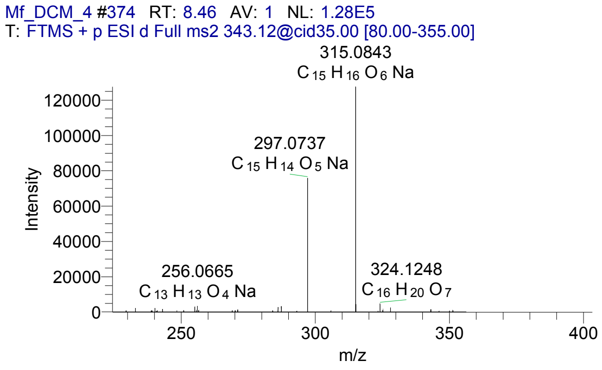

(**B**)

Figure S23. Full scan MS (**A**) and MS/MS (**B**) spectra of peak 16 of Table 1.

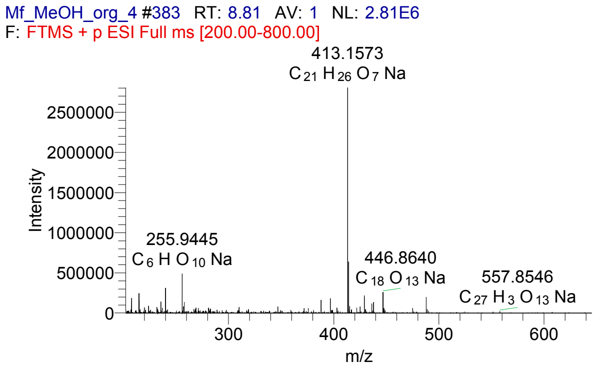

(**A**)

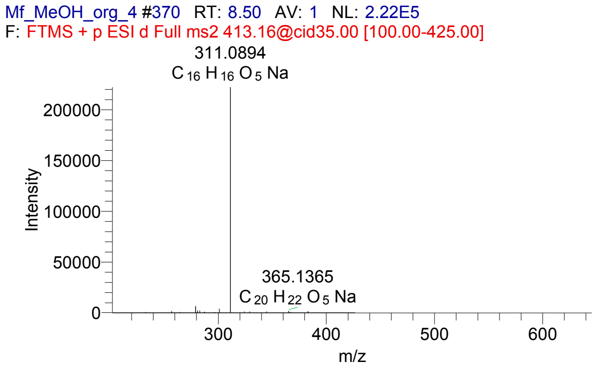

(**B**)

Figure S24. Full scan MS (**A**) and MS/MS (**B**) spectra of peak 17 of Table 1.

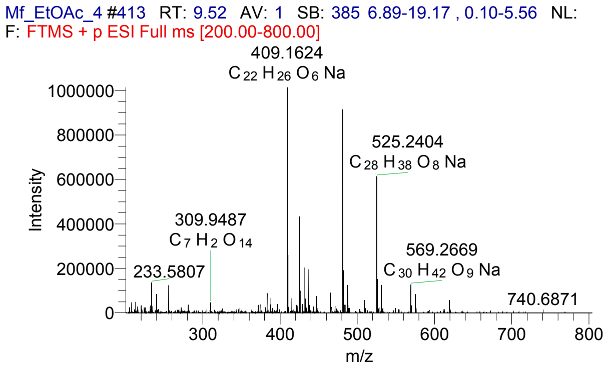

(**A**)

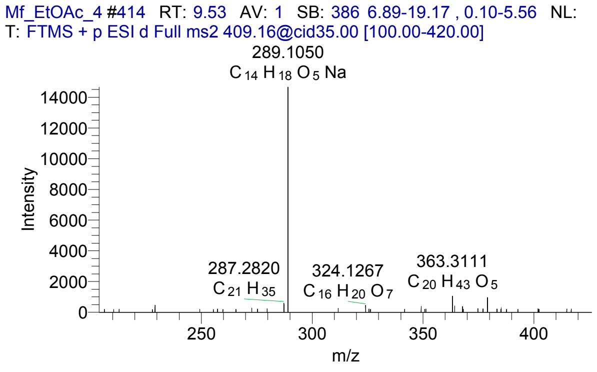

(**B**)

Figure S25. Full scan MS (**A**) and MS/MS (**B**) spectra of microfalcrin (**1**) in ESI(+).

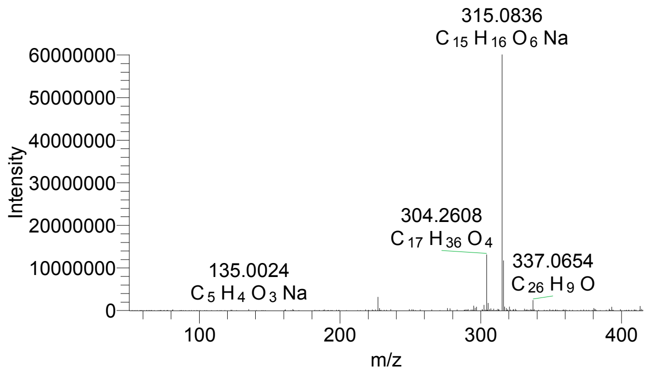

(**A**)

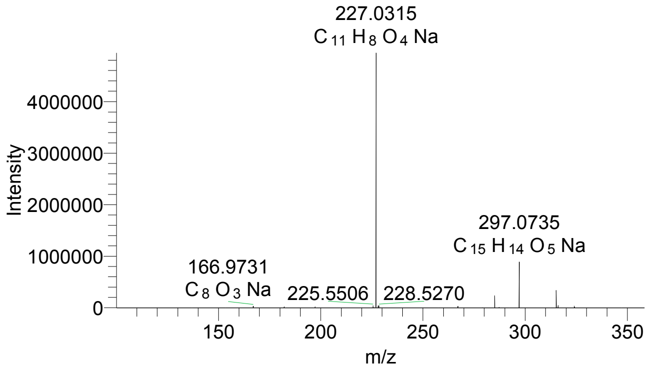

(**B**)

Figure S26. Full scan MS spectrum of microcoumaririn (**2**) in ESI(+).

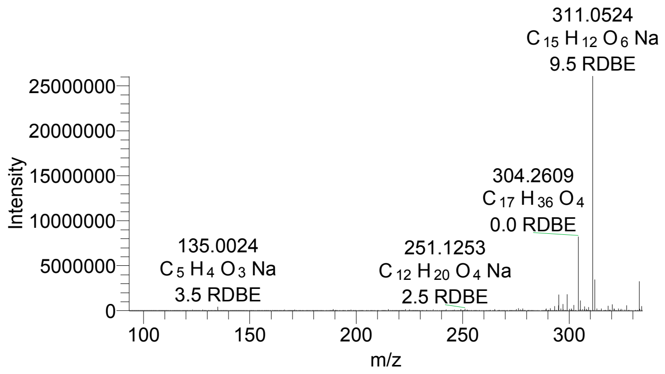

Figure S27. Full scan MS (**A**) and MS/MS (**B**) spectra of micromelosidester (**3**) in ESI(+).

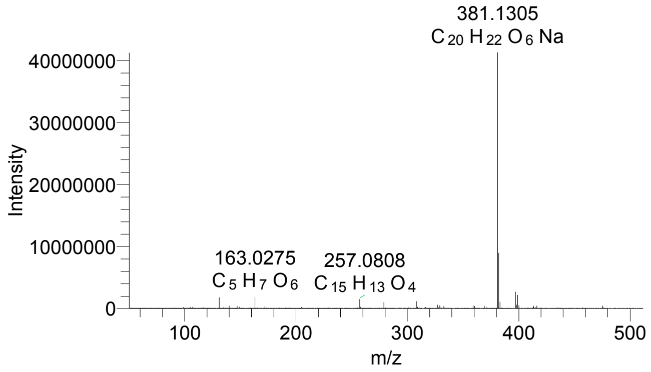

(**A**)

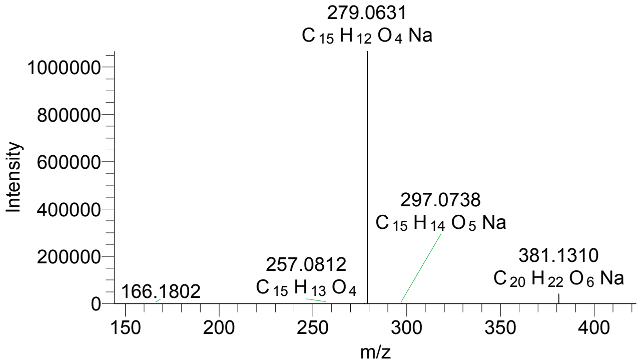

(**B**)

Figure S28. Full scan MS spectrum of micromeloside A (**4**) in ESI(+).

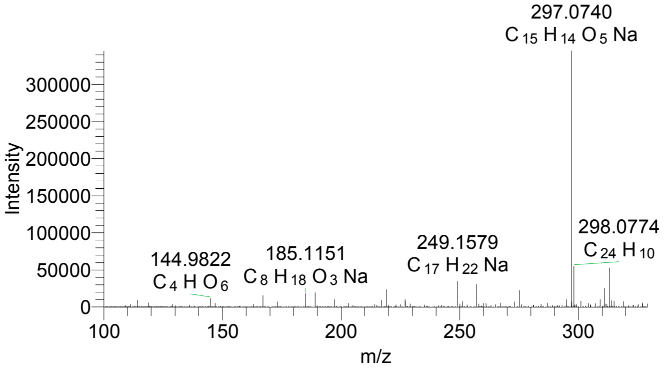

Figure S29. Full scan MS spectrum of microminutin (**5**) in ESI(+).

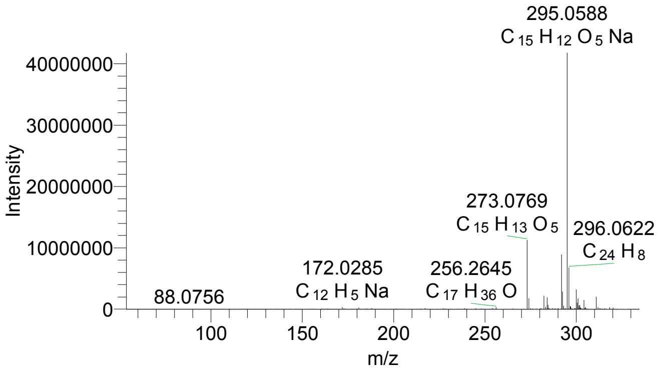

Figure S30. Full scan MS spectrum of micromelin (**6**) in ESI(+).

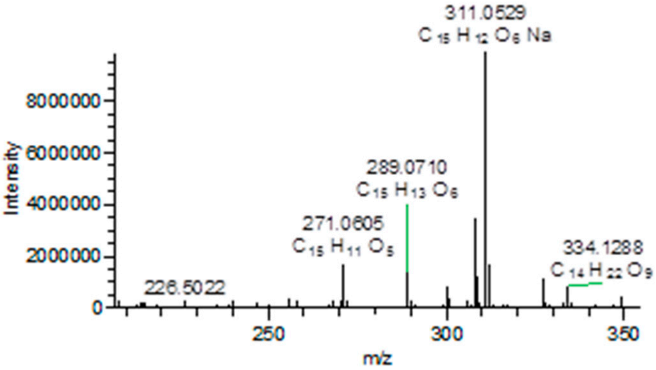

Figure S31. Full scan MS (**A**) and MS/MS (**B**) spectra of micromarin B (**7**) in ESI(+).

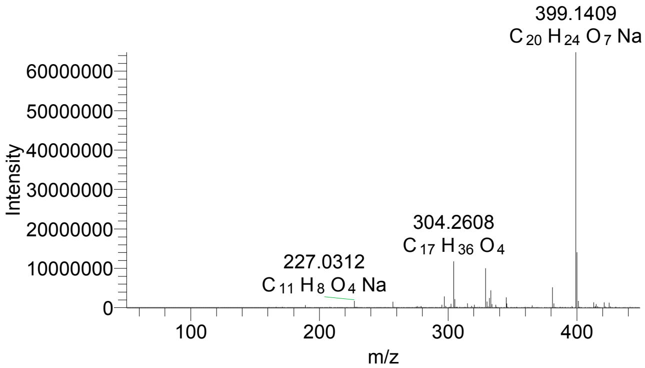

(**A**)

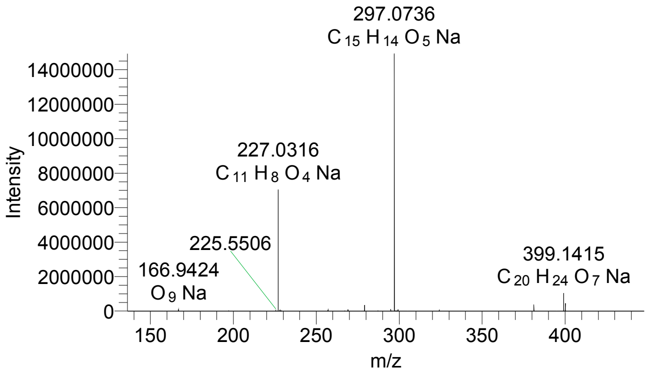

(**B**)

Figure S32. Full scan MS (**A**) and MS/MS (**B**) spectra of micromarin A (**8**) in ESI(+).

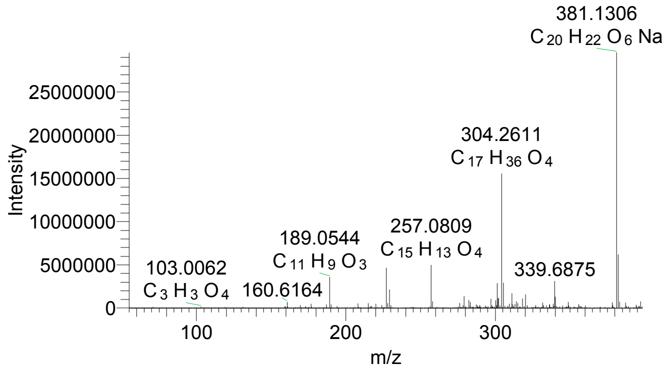

(A)

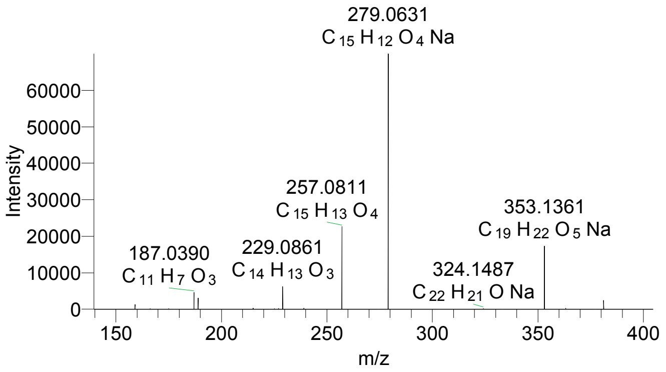

(B)

Figure S33. Percentage of nitrite oxide production relative to control (LPS-induced); Resv: Resveratrol, Mmin: Microminutin (**5**), Mmar B: Micromarin B (**7**); MmEtOAc: EtOAc extract.

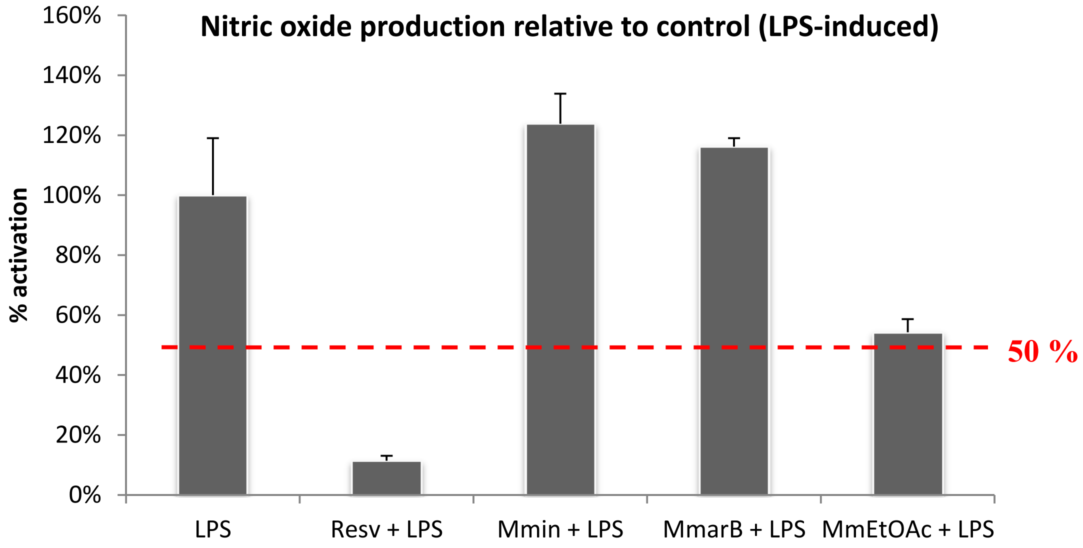

Figure S34. Percentage of NFκB induction relative to control (TNF-α treated); Resv: Resveratrol, Mmin: Microminutin (**5**), Mmar B: Micromarin B (**7**); MmEtOAc: EtOAc extract.

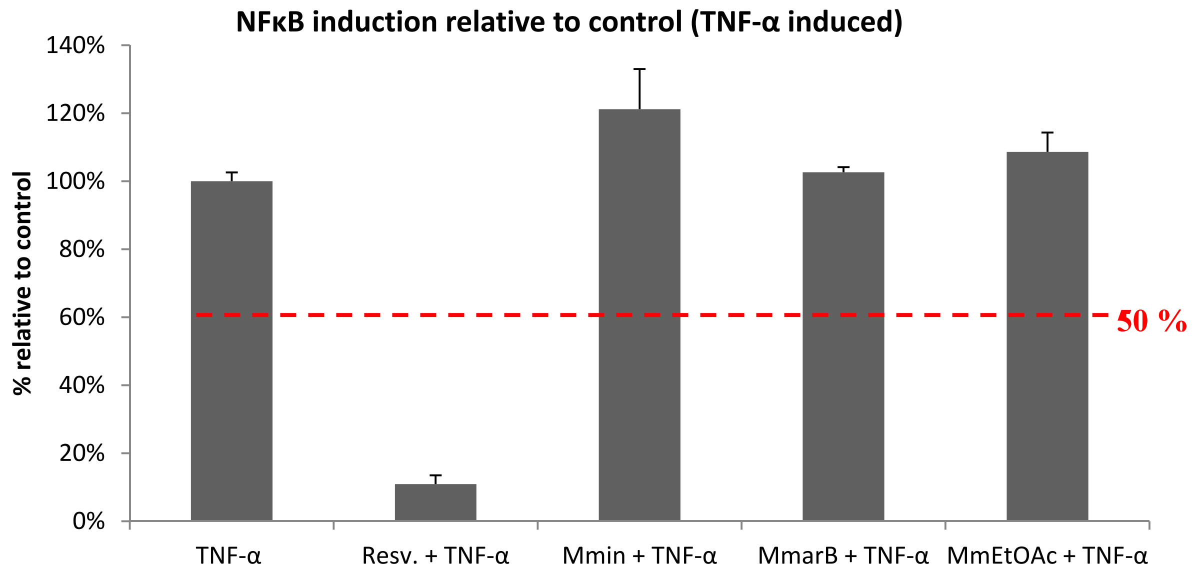

Supplement: Supplementary File 1 [file molecules-19-15042-s001.pdf]
